# Quantitatively relating brain endothelial cell–cell junction phenotype to global and local barrier properties under varied culture conditions via the Junction Analyzer Program

**DOI:** 10.1186/s12987-020-0177-y

**Published:** 2020-02-11

**Authors:** Kelsey M. Gray, Jae W. Jung, Collin T. Inglut, Huang-Chiao Huang, Kimberly M. Stroka

**Affiliations:** 1 Fischell Department of Bioengineering, University of Maryland, 8278 Paint Branch Drive, 3110 A. James Clark Hall, College Park, MD 20742 USA; 2 Biophysics Program, University of Maryland, College Park, MD 20742 USA; 3 Center for Stem Cell Biology and Regenerative Medicine, University of Maryland–Baltimore, Baltimore, MD 21201 USA; 4 Marlene and Stewart Greenebaum Comprehensive Cancer Center, University of Maryland–Baltimore, Baltimore, MD 21201 USA

**Keywords:** Blood–brain barrier, cAMP, JAnaP, Permeability

## Abstract

**Background:**

The endothelial cell–cell junctions of the blood–brain barrier (BBB) play a pivotal role in the barrier’s function. Altered cell–cell junctions can lead to barrier dysfunction and have been implicated in several diseases. Despite this, the driving forces regulating junctional protein presentation remain relatively understudied, largely due to the lack of efficient techniques to quantify their presentation at sites of cell–cell adhesion. Here, we used our novel Junction Analyzer Program (JAnaP) to quantify junction phenotype (i.e., continuous, punctate, or perpendicular) in response to various substrate compositions, cell culture times, and cAMP treatments in human brain microvascular endothelial cells (HBMECs). We then quantitatively correlated junction presentation with barrier permeability on both a “global” and “local” scale.

**Methods:**

We cultured HBMECs on collagen I, fibronectin, collagen IV, laminin, fibronectin/collagen IV/laminin, or hyaluronic acid/gelatin for 2, 4, and 7 days with varying cAMP treatment schedules. Images of immunostained ZO-1, VE-cadherin, and claudin-5 were analyzed using the JAnaP to calculate the percent of the cell perimeter presenting continuous, punctate, or perpendicular junctions. Transwell permeability assays and resistance measurements were used to measure bulk (“global”) barrier properties, and a “local” permeability assay was used to correlate junction presentation proximal to permeable monolayer regions.

**Results:**

Substrate composition was found to play little role in junction presentation, while cAMP supplements significantly increased the continuous junction architecture. Increased culture time required increased cAMP treatment time to reach similar ZO-1 and VE-cadherin coverage observed with shorter culture, though longer cultures were required for claudin-5 presentation. Prolonged cAMP treatment (6 days) disrupted junction integrity for all three junction proteins. Transwell permeability and TEER assays showed no correlation with junction phenotype, but a local permeability assay revealed a correlation between the number of discontinuous and no junction regions with barrier penetration.

**Conclusions:**

These results suggest that cAMP signaling influences HBMEC junction architecture more than matrix composition. Our studies emphasized the need for local barrier measurement to mechanistically understand the role of junction phenotype and supported previous results that continuous junctions are indicative of a more mature/stable endothelial barrier. Understanding what conditions influence junction presentations, and how they, in turn, affect barrier integrity, could lead to the development of therapeutics for diseases associated with BBB dysfunction.

## Introduction

Endothelial cell–cell junctions, especially within the blood–brain barrier (BBB), are extremely important for maintaining normal physiological processes. They regulate numerous cell functions (e.g., migration, proliferation) and paracellular transport across the barrier, such that decreased junctional protein is associated with dysregulated transport and leaky vasculature [1, 2]. Despite the numerous diseases associated with altered junctions, [3] the mechanisms regulating junctional proteins remain relatively understudied, largely due to the lack of efficient techniques to quantitatively assess their presentation at sites of cell–cell adhesion. Furthermore, the specific influence of different junction phenotypes on BBB properties remains elusive. We have recently developed the Junction Analyzer Program (JAnaP) to quantify the phenotypic junction presentation within the endothelium [4, 5]. Here, we used the JAnaP to study the influence of junction phenotype on human brain microvascular endothelial cell (HBMEC) barrier properties.

One difficulty in studying the BBB in vitro is the challenge associated with recapitulating the in vivo HBMEC properties [6], such as the overexpression of the tightly structured network of endothelial junctions. One technique to improve tight junction formation and barrier properties, evidenced by junction immunostaining, Transwell permeability studies, and transendothelial electrical resistance (TEER) measurements, is the co-culturing of endothelial cells (ECs) with neural cells (e.g., astrocytes) or their conditioned medium [7–12]. This not only improved BBB properties but also provided insights into the roles of biochemical and physical contacts of brain ECs with other cells present in the in vivo microenvironment. Another biomimetic approach has been the use of different matrix proteins that (at least partially) recapitulate the in vivo basement membrane or the brain microenvironment. The basement membrane is known to have an important role in maintaining vascular function [13]. As such, is it unsurprising that constituents of this matrix (i.e., fibronectin, collagen type IV, and laminin; or combinations of the three) are reported to elevate TEER values relative to type I collagen in porcine brain capillary ECs, [14] and promote adhesion and spreading of iPSC-derived brain ECs [15]. Additionally, hyaluronic acid is a primary component of the brain microenvironment, [16] and has been shown to induce tube formation in a mouse-derived brain capillary EC line [17]. A mixture of hyaluronic acid and gelatin is reported to improve cell spreading of endothelial progenitor cells and human umbilical vein ECs (HUVECs) [18] and has been used for in vitro models of the BBB [5, 19]. Another approach towards improving the brain EC phenotype is the activation of cyclic 3′-5′-adenosine monophosphate (cAMP)-dependent protein kinase (PKA) via cAMP, dexamethasone, or hydrocortisone, which is linked with improved barrier function [10, 20–23]. Specifically, we and others have shown that 8-(4-chlorophenylthio) adenosine-3′,5′-cyclic monophosphate sodium salt (CPT-cAMP) and 4-(3-butoxy-4-methoxybenzyl) imidazolidin-2-one (RO-20-1724) decrease permeability and increase tight junctions in various EC types [4, 24–27]. Furthermore, barrier confluency and maturity are also reported to influence junction presentation within the endothelium [28, 29].

Here, our goal was to probe the influence of junction phenotype on HBMEC barrier properties using the JAnaP. We first investigated different in vitro factors to identify conditions driving altered states of junction presentation in HBMEC monolayers. Specifically, we studied the effects of substrate protein coating, culture time, and treatment with cAMP supplements. We then used those parameters to evaluate barrier permeability and tightness (via TEER) as a function of junction phenotype.

## Methods

### Cell culture

Primary HBMECs were purchased from Cell Systems (ACBRI 376) and cultured as previously described [5]. Briefly, cells were seeded into flasks coated with 0.1% gelatin, and cells were cultured in RPMI-1640 medium supplemented with 20% FBS, 1% Pen/Strep, 2 mM l-glutamine (Thermo Fisher Scientific), 30 μg/ml endothelial cell growth supplement (ECGS) (Millipore Sigma), and 100 μg/ml heparin (Millipore Sigma) at 37 °C, with 5% CO2 and 50% humidity. Cells arrived in our lab mycoplasma-free upon receipt from Cell Systems (according to the certificate of analysis), expanded, and used for experiments within passages 7–10. Cultures were tested after approximately 6 months and found to be mycoplasma-free using the MycoAlert PLUS Mycoplasma Detection Kit (Lonza, LT07-701).

### Substrate Coating and Experimental Conditions

On Day 0, glass bottom 24-well plates (Greiner Bio-One, 662892) were coated with 175 μl of one of the following: 100 µg/ml collagen I (CN) (Sigma Aldrich, C3867), 100 µg/ml fibronectin (FBN) (Sigma Aldrich, F2006), 100 µg/ml collagen IV (CIV) (Sigma Aldrich, C6745), 100 µg/ml fibronectin + 100 µg/ml collagen IV + 2 µg/cm^2^ laminin (Fbn:CIV:L or F:C:L), or 0.4% thiol-modified hyaluronan: 0.4% thiol-modified gelatin (HA/Gtn) (ESI-BIO, GS313) for 30 min at 37 °C, or 2 µg/cm^2^ laminin (LN) (Sigma Aldrich, L4544) for 60 min at 37 °C. All constituents were resuspended per the manufacturer’s instructions, then diluted to the respective concentration in Dulbecco’s Phosphate-Buffered Saline, 1× with calcium and magnesium (Corning, 21-030-CV) (PBS). After coating the surface, the excess solutions were removed, the wells were rinsed with 37 °C PBS, 500 µl of warm HBMEC medium was added to each well, and the plate was incubated at 37 °C until HBMEC seeding (approximately 20 min). Cells were seeded (5 × 10^4^ cells/cm^2^, 9.5 × 10^4^ cells/well), then 500 µl of warm medium was additionally added to each well, and the cells were cultured for 2, 4, or 7 days. Samples were treated with medium containing cAMP supplements: 250 μM 8-CPT-cAMP (Abcam, ab120424) and 17.5 μM RO-20-1724 (Tocris Bioscience, 0415), for 1, 3, or 6 days, or control HBMEC medium. These supplements are routinely used in EC culture to improve junction localization and barrier properties [4, 30–33]. For all experiments, the medium was first changed the day after cell seeding, then again on Days 3, 4, and 6 for the respective culture lengths. On the final day of culture, cells were fixed as described below in “Immunostaining” section. Three biological replicates were performed for each experiment. A summary of each culture condition is presented as Figs. 2a, 3a and 4a, respectively.

### Immunostaining

HBMECs were rinsed with 37 °C PBS and fixed with 1% formaldehyde in PBS (ThermoFisher Scientific, BP531) for 20 min. Note that all steps were performed under gentle rocking. Samples were washed three times, 5 min each, with room temperature PBS, then permeabilized for 5 min with 0.25% TritonX-100 (Sigma-Aldrich) in PBS. The wash steps were repeated then the samples were blocked for 1 h at room temperature with 2% goat serum (Abcam) in PBS. Primary antibodies against ZO-1 (rabbit polyclonal IgG, ThermoFisher Scientific, 61-7300, 1:500) and VE-cadherin (mouse monoclonal IgG, Santa Cruz, sc-9989, 1:50) in 2% goat serum in PBS were added to the cells overnight at 4 °C. The next day, the wash and blocking steps were repeated. Secondary antibodies goat anti-rabbit Alexa Fluor 488 (Abcam, ab150077, 1:100) or goat anti-rabbit Alexa Fluor 568 (ThermoFisher Scientific, A-11011), and/or goat anti-mouse Alexa Fluor 568 (ThermoFisher Scientific, A-11004, 1:100), and Hoechst (ThermoFisher Scientific, H3570, 1:2500 or 4 μg/ml), were then added to the sample in PBS for 1 h at room temperature. The wash steps were again repeated prior to imaging. For claudin-5 staining (rabbit polyclonal IgG, Abcam, ab15106, 1:200), cells were instead fixed with 100% ice cold methanol (Sigma Aldrich) for 10 min and blocked with 2% goat serum in PBS containing 0.3% TritonX-100 for 1 h at room temperature.

### Junction analysis

**Fig. 1:**
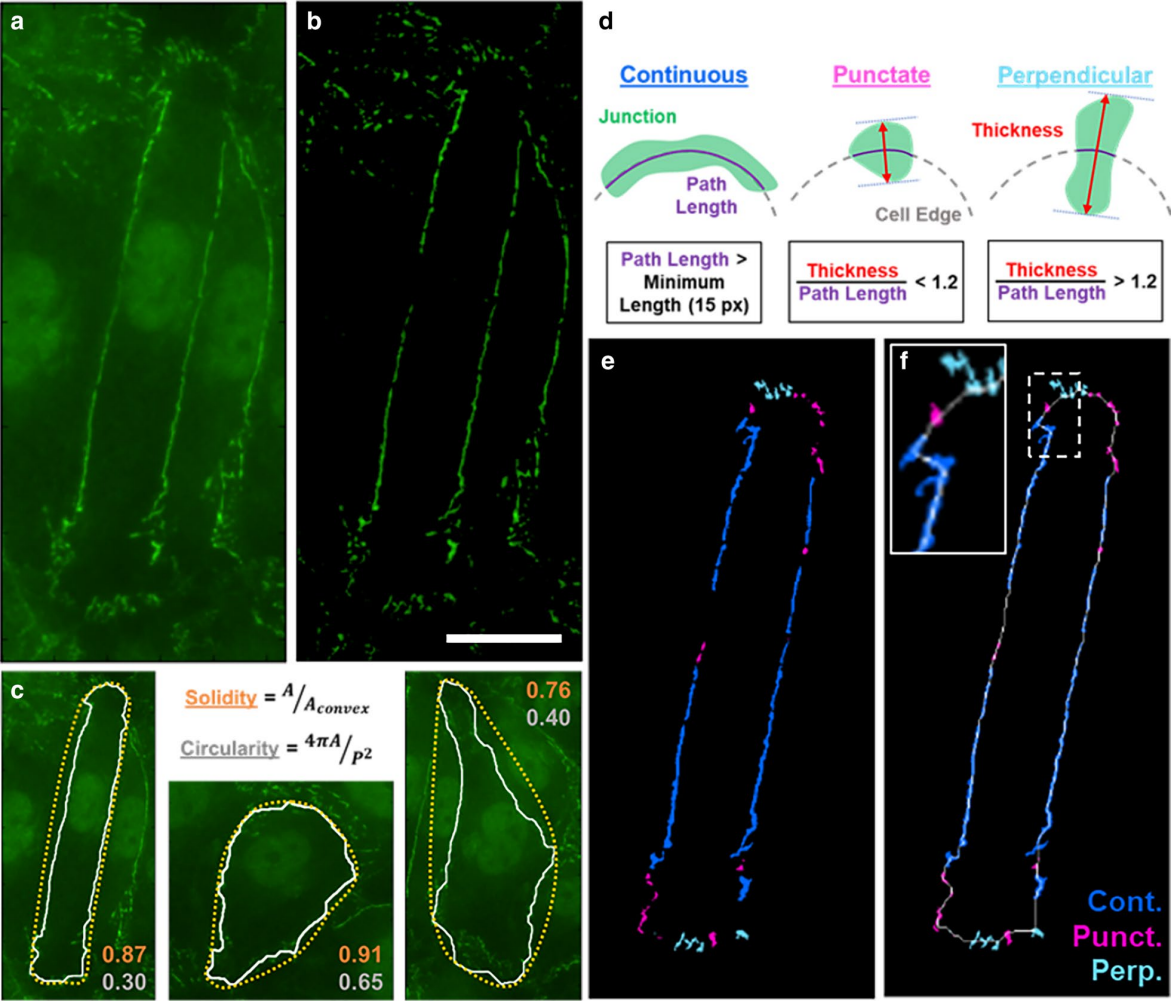
JAnaP workflow. **a** Example cell of interest within a monolayer immunostained for ZO-1. **b** Isolated cell junctions after the filtering and threshold value has been applied. **c** Example cells depicting solidity (orange) and circularity (gray) calculations, where A and P are the cell area and perimeter (white outline), and Aconvex is the convex area (yellow outline). **d** Criteria for junction categorization to differentiate between continuous (cont., blue), punctate (punct., magenta), and perpendicular (perp., light blue) junctions. **e**–**f** Categorized junctions for the cell of interest, where (**e**) also depicts the perimeter of the cell (thin white line) and a cropped image of the dotted white region to improve visibility of the junctions (scale bar = 20 μm)

Junction presentation was quantified using the Junction Analyzer Program (JAnaP) [4], available for download at https://github.com/StrokaLab/JAnaP. A simplified workflow of the JAnaP is presented in Fig. 1. Briefly, cells whose perimeter was completely visible within each image were traced via “waypointing”. For ZO-1 and VE-cadherin quantification, waypointing was performed on the images of ZO-1 (captured in the green fluorescent channel, A488), and the waypoints were projected onto the images of VE-cadherin (captured in the red fluorescent channel, A568), as previously described in [5]. For claudin-5 quantification, the cells were traced using the red fluorescent channel (i.e., VE-cadherin immunostaining) and the waypoints were projected onto the green fluorescent channel (i.e. claudin-5 immunostaining), as the cell-edge was more visible in the images of VE-cadherin versus claudin. Note that the images reflect pseudo-color imaging. Threshold values of 15, 5, and 5, were applied to isolate the ZO-1, VE-cadherin, and claudin-5 junctions, respectively. Note that instructions on how to identify threshold values is described in the supplement of [4] and in the JAnaP User-Guide available using the link above. In short, different threshold values were manually investigated for several cells representing the range of brightness throughout the sample images to identify a threshold value that appropriately isolates the junction pieces for that protein. The cell morphological parameters (e.g., area, solidity, circularity) were then calculated, as well as the percent of the cell edge presenting continuous, punctate, or perpendicular junction. Junction phenotypes were classified based on the length of the junction piece that coincides with the cell path (> 15 pixels for continuous junction) and the relative aspect ratio with respect to the cell path (> 1.2 for perpendicular junction, otherwise punctate), which serve as constant parameters when using the JAnaP. Note that discontinuous junctions refer to the sum of the punctate and perpendicular junction results.

### Transwell permeability assay

For the Transwell permeability assay, HBMECs were seeded (5 × 10^4^ cells/cm^2^, 1.6 × 10^4^ cells/well) into Transwell inserts (Falcon, 24 well format, 1.0 µm pore size) that had been coated with 100 µg/ml FBN for 30 min at 37 °C. The next day, the medium was changed to control medium or cAMP-medium. On the following day, solutions of 1 mg/ml FITC-Dextran (70 kDa, Sigma-Aldrich) were prepared in the respective medium formulations. Each Transwell was moved to a new well containing 800 µl of fresh medium and the top well was replaced with 400 µl of the dextran-medium. After 30 min at 37 °C, the medium in the bottom well was collected, and the fluorescence was measured using a BioTek Synergy Neo2 plate reader (Excitation/Emission: 492/518 nm, Gain: 65). A standard curve was used to calculate the mass of dextran within the sample and the apparent permeability coefficient (P_app) was calculated as previously described by Tominaga et al., [34]:

$$P\_app = \left( {V \cdot \left[ {C_{abluminal} } \right]} \right) \cdot A^{ - 1} \cdot \left[ {C_{luminal} } \right]^{ - 1} \cdot t^{ - 1} \left[ = \right] \;{\text{cm}}/{\text{s}}$$

where, V is the volume of the abluminal chamber, A is the surface area of the membrane, [C_abluminal_] is the measured abluminal dextran concentration at time, t, and [C_luminal_] is the initial luminal dextran concentration added. The inserts were then fixed and stained as described in the “Immunostaining” section above. For imaging, the membranes were removed from the inserts using an X-acto knife and sandwiched between two coverslips, luminal-side down. Three biological replicates were performed for this experiment.

### Local (XPerT) permeability assay

**Fig. 2:**
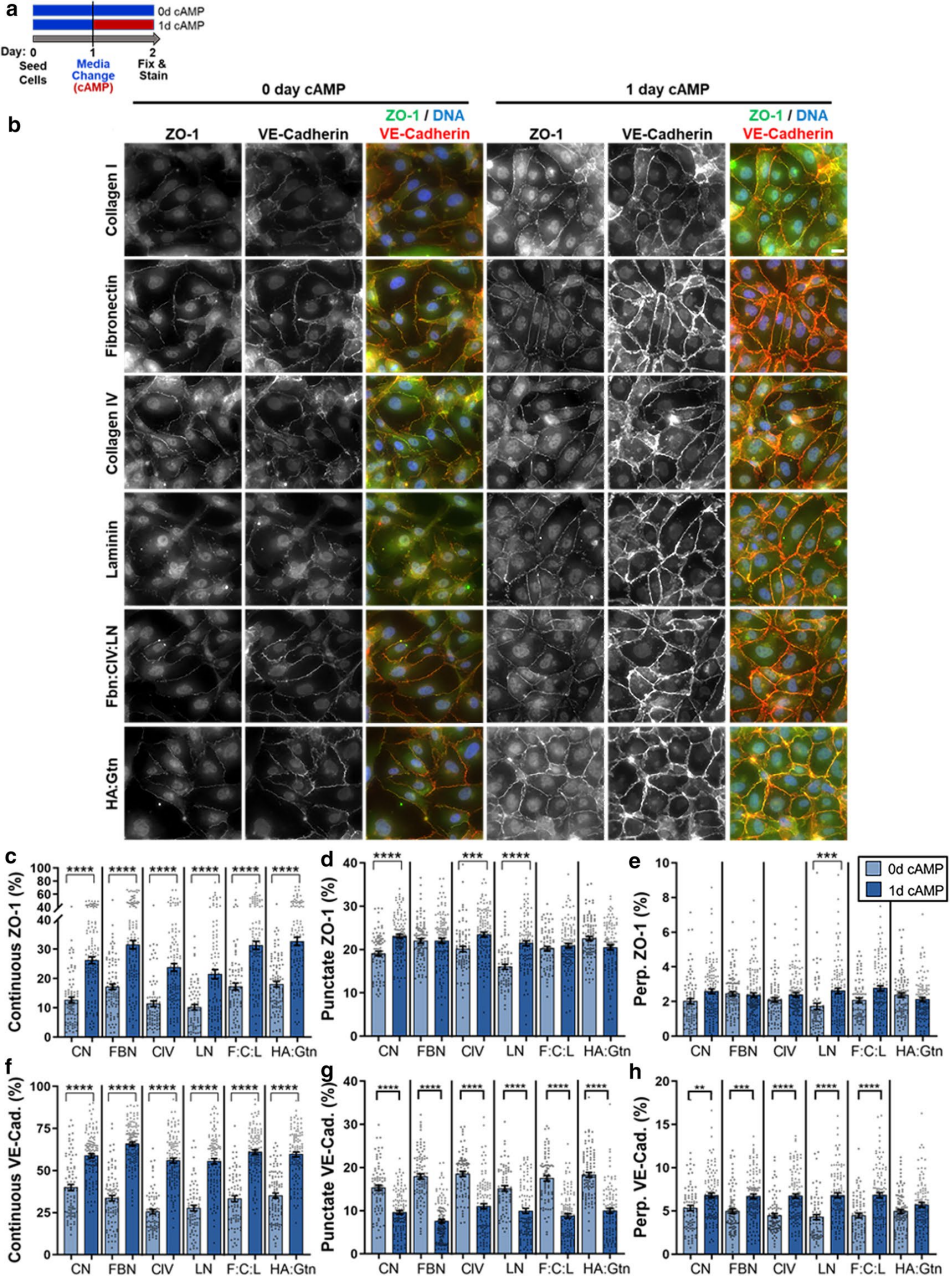
Immunofluorescence images and junction phenotype analysis for 2-day culture. **a** Schematic representing treatment schedule for 2-day experiment. **b** HBMECs on 6 substrate coatings, cultured for 2 days with and without cAMP treatment, stained for ZO-1 (green), VE-cadherin (red), and DNA (blue). (scale bar = 20 μm) Edge presentation of continuous (**c**, **f**), punctate (**d**, **g**), and perpendicular (**e**, **h**) junctions for ZO-1 and VE-cadherin, respectively. 72 ≤ N ≤ 125, where N is the number of cells. The Kruskal–Wallis test with a Dunn’s multiple comparison test was used to calculate significant differences, where *p < 0.05, **p < 0.01, ***p < 0.001, and ****p < 0.0001. See Additional file 1: Table S2 for statistical comparison between each protein coating

To visualize areas of monolayer leakiness, and to correlate them with junction phenotype, we adapted the XPerT permeability assay developed by Dubrovskyi et al. [35]. Here, however, FBN was biotinylated (B-FBN) using EZ-Link NHS-LC-LC-Biotin (ThermoFisher Scientific, 21343) according to the manufacturer’s instructions. B-FBN was then adsorbed onto glass bottom 24-well plates overnight at 4 °C. Excess protein was then removed, the wells were rinsed with PBS, and 500 µl of warm HBMEC medium was added to each well. The plate was incubated at 37 °C until HBMEC seeding (approximately 20 min). After the cells were seeded (5 × 10^4^ cells/cm^2^, 9.5 × 10^4^ cells/well), 500 µl of warm medium was additionally added to each well, and the cells were cultured per the 2-day experiment in Fig. 2a. Immediately before fixing, samples were treated with 50 µg/ml FITC-avidin (ThermoFisher Scientific, A821) for 3 min to enable FITC-avidin binding to the underlying b-FBN at permeable sites of the monolayer. The samples were then fixed and stained for ZO-1 and VE-cadherin per the “Immunostaining” section above. Three biological replicates were performed for each junction protein.

### Local permeability analysis

To analyze the results of the local permeability assay, two primary steps were performed. An example monolayer image is presented in Additional file 1: Figures S1 and S2 to depict each step. First, image processing of the green-channel images of the bound FITC-avidin was performed in ImageJ. To do this, every image was converted to 8-bit and a threshold intensity value of 240 was applied to create a binary image showing the presence or absence of a permeated region (PR). The second step was to process the red-channel junctional protein images using the JAnaP. This analysis differed from single cell analysis using the JAnaP, since every single cell border was waypointed, regardless of whether the entire cell was present in the image. The JAnaP-associated Jupyter Notebook [4] was then used to generate several images of the categorized junctions, in some cases, overlaid them onto the PR threshold images. For the quantification of this assay, several parameters were studied, as outlined below.

#### PR categorization

PRs were categorized as Uni, Bi, Tri, Quad, or Multi, depending on the number of cells the PR was associated with (1, 2, 3, 4, 5+, respectively) (Additional file 1: Figure S1). To quantify PR area, the Analyze Particles function in ImageJ was used on the PR threshold images. Images showing the cell edges on top of the PRs were used to manually identify the number of cells that each PR was adjacent to. Five images from each of the 3 trials were measured, and the average count of each PR category per image was calculated. The PR area measurements were averaged over all PRs within the respective category.

#### Junction analysis along PR length

To calculate the percent and count of junctions along the cell perimeters coinciding with PRs, images depicting the JAnaP-analyzed junctions overlaid onto the threshold images were used (Additional file 1: Figure S1). These overlaid images were manually traced in ImageJ using the segmented line tool. Importantly, only PRs greater than 400 pixels^2^ were included in this analysis. For each PR, the length of the cell path(s) overlapping the PR was manually traced to calculate the PR length. Then, the number and length of each junction type was subsequently summed. The difference was taken to be the length of the no junction regions. The summed length of each junction type divided by the PR length was taken to be the % Junction Along the PR Path. Three images from each of the 3 trials were measured, with the values calculated on a per PR basis.

#### Co-localization analysis

For the co-localization analysis, the JAnaP-associated Jupyter Notebook was used to generate junction-categorized images that presented all the junctions for each category within a given image, on a black background without the cell path (Additional file 1: Figure S2). These images were uploaded into ImageJ, converted to 8-bit, and a threshold was applied to isolate the junctions. A selection was then created to measure the total area of each junction type present within the image (A_total). Next, the PR threshold images were again uploaded into ImageJ. A selection was created to isolate the PRs and was used as a mask applied to each junction image. The junctions present outside of the masked PR region were removed, leaving only the junction pieces corresponding to the PRs remaining. Another selection was created to measure the area of each junction type that corresponded with PRs in the image (A_PR). The % Co-localization was taken as (A_PR/A_total)*100 for each junction type.

### Microscopy

All samples were imaged using a 60× oil objective on an inverted IX83 Olympus microscope and Olympus cellSens Software. For fixed-cell epifluorescence microscopy, images were simultaneously collected using the red, green, and blue filters. Images within the manuscript have been enhanced via ImageJ for improved visualization.

### Statistical analysis

All statistical analysis and graph generation was performed using GraphPad Prism 8. For each data set, a D’Agostino-Pearson normality test was used to identity the normality of the data. If the data was normal, a one-way ANOVA with a Tukey’s multiple comparison post hoc test was performed. More frequently, the data was non-normal, in which case the non-parametric Kruskal–Wallis ANOVA with Dunn’s multiple comparison post hoc testing was performed instead. For instances where only two groups were compared, a Mann–Whitney test was used. A linear regression was used to compare the junction presentation with global permeability. No statistical significance (ns) was determined using p > 0.05, and statistical significance was indicated as *p ≤ 0.05, **p ≤ 0.01, ***p ≤ 0.001, ****p ≤ 0.0001. Errors bars represent standard error of the mean. All data represents pooled values from three independent trials.

## Results

### cAMP supplements increase continuous ZO-1 and VE-cadherin junctions, independently of substrate coating

Figure 2 presents HBMECs cultured for 2 days with and without cAMP supplements on six different matrix constituents. While almost no differences in cell area, solidity, or circularity were observed between each substrate coating (Additional file 1: Figure S3 and Table S1), differences in junctional protein presentation were found. In general, CIV and LN induced lower junctional protein presentation compared to the other substrate coatings (Additional file 1: Table S2). As expected, the addition of cAMP supplements significantly improved barrier architecture. Increases in continuous junction were observed with cAMP treatment for every condition for both ZO-1 and VE-cadherin (Fig. 2), with the greatest presentation observed on FBN (though, F:C:L and HA:G induced similar coverage). For ZO-1, cAMP increased punctate junctions only on CN and CIV, and increased both punctate and perpendicular junctions only on LN. Discontinuous VE-Cadherin, on the other hand, was significantly influenced by cAMP supplements. Specifically, cAMP treatment decreased punctate VE-cadherin and increased perpendicular VE-cadherin for nearly every condition except HA:Gtn in which perpendicular junctions remained unchanged. Note that the total junction coverage (i.e. sum of continuous, punctate, and perpendicular junctions) for the conditions of this and following experiments is presented in Additional file 1: Figure S4.

### Extending cell culture requires increased cAMP treatment for similar junction coverage

We next investigated the effects of extending cell culture and cAMP treatment time to probe the ability of these parameters to further increase junction coverage. Since we observed minimal differences between substrate protein coatings, here we focused our results on FBN. The immunofluorescence images, cell morphology, and junction presentation results on all other substrate coatings can be found in Additional file 1: Figures S5–S10 and Tables S3–S6.

**Fig. 3:**
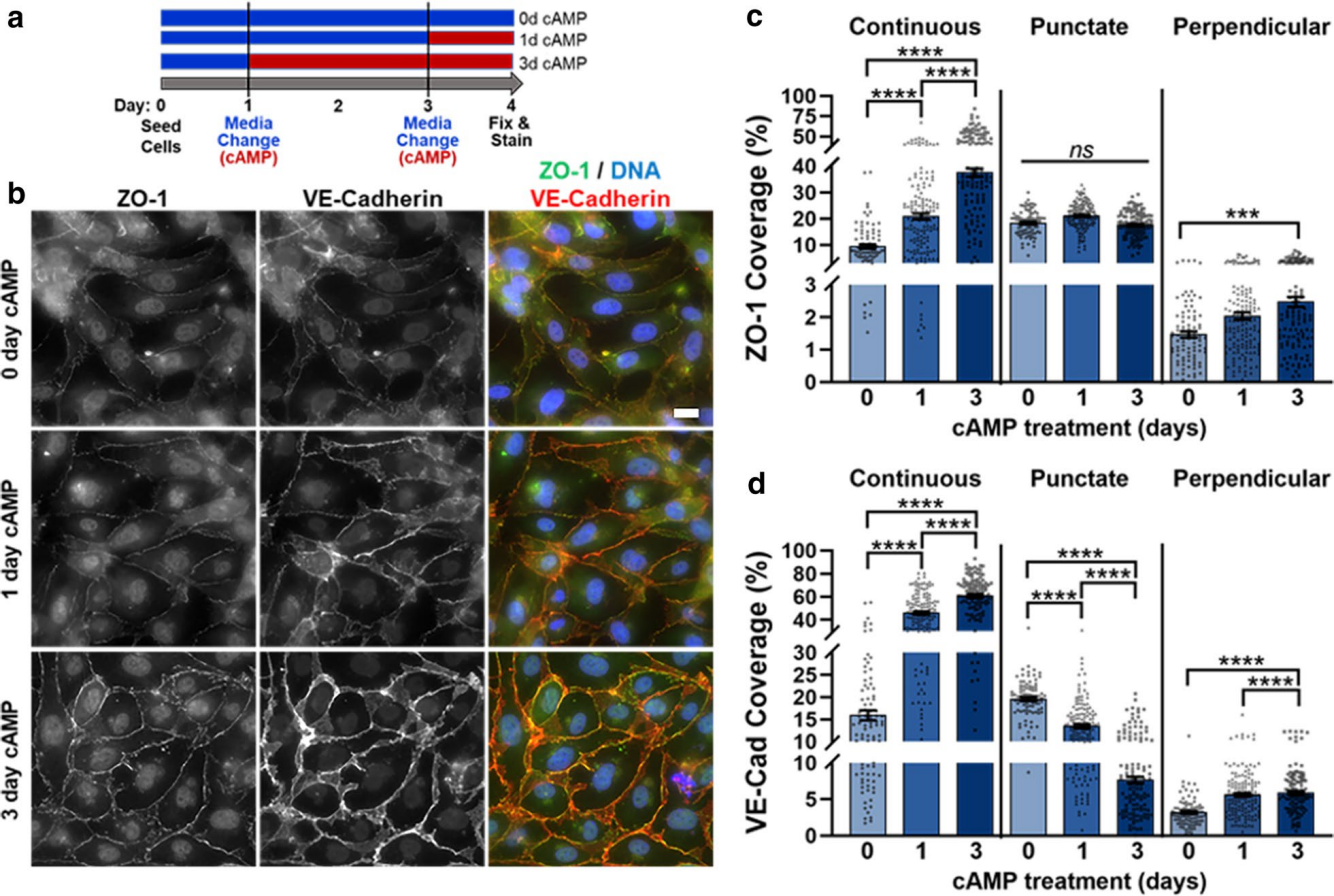
Immunofluorescence images and junction phenotype analysis for 4-day culture. **a** Schematic representing treatment schedule for 4-day experiment. **b** HBMECs on Fbn, cultured for 4 days with 0, 1, or 3 days of cAMP treatment, stained for ZO-1 (green), VE-cadherin (red), and DNA (blue). (scale bar = 20 μm) Edge presentation of continuous, punctate, and perpendicular junctions for (**c**) ZO-1 and (**d**) VE-cadherin. 87 ≤ N ≤ 145, where N is the number of cells. The Kruskal–Wallis test with a Dunn’s multiple comparison test was used to calculate significant differences, where ns = p > 0.05, ***p < 0.001, and ****p < 0.0001

First, we studied the effects of extending the time in cell culture to 4 days. Figure 3 presents HBMECs cultured for this length of time with 0, 1, or 3 days of cAMP supplement treatment. No changes in cell circularity or solidity were observed regardless of condition or treatment, and generally insignificant changes in cell area were observed, except on CIV and HA:Gtn where 1d cAMP generated led to smaller cell areas than the 3d treatment group (and 1d cAMP in the case of HA:Gtn) (Additional file 1: Figure S6). Interestingly, though, cells were smaller in area after 4 days of cultures compared to cells cultured for 2 days, except for 4D/3d cAMP groups where increased area was observed. Increased cAMP treatment increased both continuous and perpendicular ZO-1 and VE-cadherin (Fig. 3, Additional file 1: Figure S7, and Table S4). The greatest continuous junction presentation was observed with 3d cAMP, where approximately 38% and 61% of the cell edge was covered for ZO-1 and VE-cadherin, respectively. These coverage values were similar to those observed in HBMECs cultured for 2 days with 1d cAMP, suggesting increased culture time required increased cAMP treatment to reach comparable junction presentation. Punctate junctions, on the other hand, displayed different responses for ZO-1 and VE-cadherin. While no change in punctate ZO-1 was observed, punctate VE-cadherin decreased with increased cAMP treatment.

**Fig. 4:**
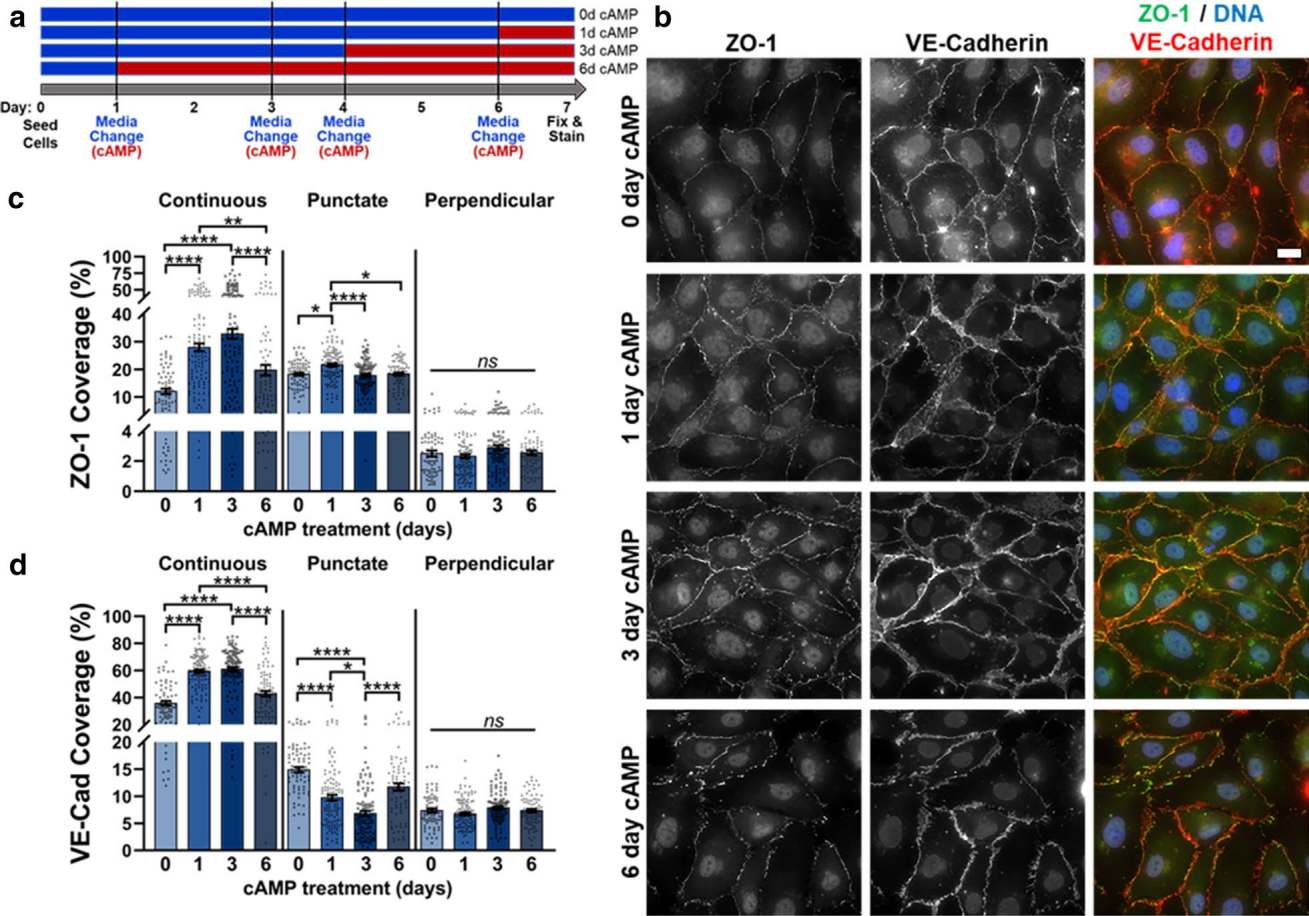
Immunofluorescence images and junction phenotype analysis for 7-day culture. **a** Schematic representing treatment schedule for 7-day experiment. **b** HBMECs on Fbn, cultured for 7 days 0, 1, 3, or 6 days of cAMP treatment, stained for ZO-1 (green), VE-cadherin (red), and DNA (blue). (scale bar = 20 μm) Edge presentation of continuous, punctate, and perpendicular junctions for **c** ZO-1 and **d** VE-cadherin. 74 ≤ N ≤ 115, where N is the number of cells. The Kruskal–Wallis test with a Dunn’s multiple comparison test was used to calculate significant differences, where ns = p > 0.05, *p < 0.05, **p < 0.01, and ****p < 0.0001

Next, we studied the effects of extending cell culture to 7 days with 0d, 1d, 3d, or 6d cAMP treatment (Fig. 4 and Additional file 1: Figure S8). Again, cell circularity and solidity generally remained consistent regardless of cAMP treatment time, except on F:C:L, where increased cAMP treatment led to more solid and circular cells (Additional file 1: Figure S9). On the other hand, cells with the largest cell area were mostly observed with 6d cAMP. Notably, the cell area with 7-day culture was comparable to the size of cells cultured for 2 days, versus 4-day culture where smaller cells were observed. Continuous ZO-1 increased with increased cAMP treatment up to 3d, while continuous VE-cadherin increased with 1d of cAMP treatment and remained at the same level with 3d cAMP treatment (Fig. 4, Additional file 1: Figure S10, and Additional file 1: Table T6). For both junction proteins, however, 6d of cAMP treatment led to a significant decrease in continuous junction presentation, to values comparable to that at 0d treatment. While no change in perpendicular junctions was observed, punctate ZO-1 increased with 1d cAMP treatment, and punctate VE-cadherin decreased with increased cAMP treatment up to 3d, then spiked up with 6d cAMP treatment. Cumulatively, the greatest total protein coverage observed during 7-day culture on FBN was with 3d cAMP treatment, with approximately 54% of the cell edge covered by ZO-1 and 76% by VE-cadherin (Additional file 1: Figure S4). These values were comparable to the total coverage observed during 4-day culture with 3d cAMP treatment, and 2-day culture with 1d cAMP treatment. This suggests that increased cAMP treatment is needed to maintain ZO-1 and to a lesser extent, VE-cadherin, with increased culture time. Importantly, there seems to be a limit to this trend since a decrease in continuous junctions (and an increase in punctate VE-cadherin) was observed with 6d cAMP treatment. Furthermore, these studies suggest that FBN may be a suitable matrix for HBMEC culture to induce varying degrees of ZO-1 and VE-cadherin coverage by varying cAMP treatment.

### Increased cell culture time increases continuous claudin-5 junctions

**Fig. 5:**
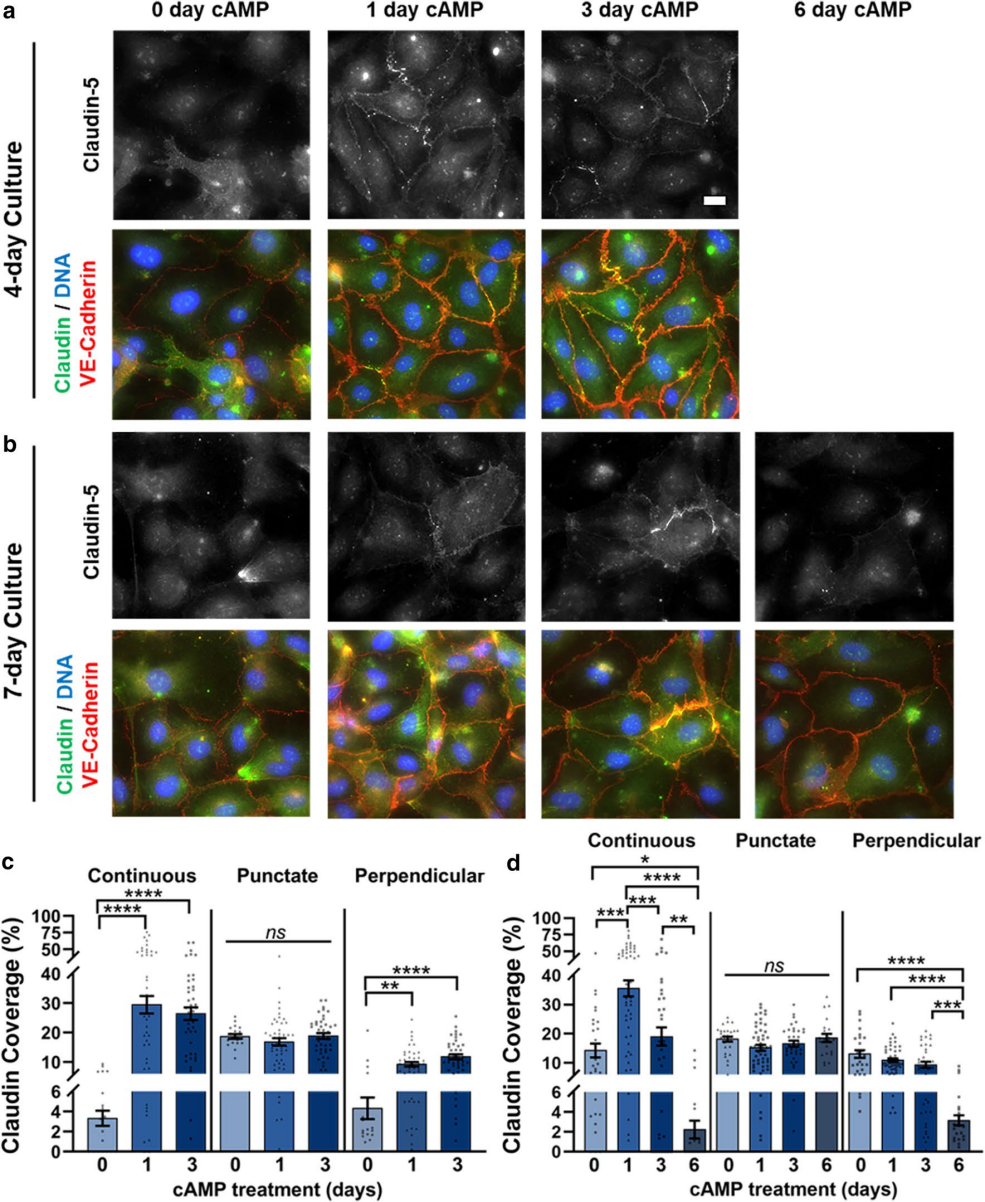
Immunofluorescence images and junction phenotype analysis for claudin-5. HBMECs on Fbn, cultured for **a** 4-days and **b** 7-days, with 0, 1, 3, or 6 days of cAMP treatment, stained for claudin-5 (green), VE-cadherin (red), and DNA (blue) (scale bar = 20 μm). Edge presentation of continuous, punctate, and perpendicular junctions for claudin-5 for **c** 4-day (19 ≤ N ≤ 47) and **d** 7-day culture (21 ≤ N ≤ 52), where N is the number of cells. The Kruskal–Wallis test with a Dunn’s multiple comparison test was used to calculate significant differences, where ns = p > 0.05, *p < 0.05, **p < 0.01, ***p < 0.001, and ****p < 0.0001

Since tight junctions are known to assemble after adherens junctions, [36] we next investigated the effects of increased cell culture and cAMP treatment time on the phenotypic presentation of tight junction protein claudin-5 (Fig. 5). First, we cultured HBMECs for 4 days on FBN coating with 0d, 1d, or 3d cAMP treatment. We observed increased edge-localization of claudin with cAMP treatment, in the form of continuous and perpendicular junctions, independent of cAMP treatment time, with no change in punctate junction presentation (Fig. 5a, c). Next, we extended the culture time to 7 days, and observed minimal claudin presentation with 6d cAMP treatment, in line with our observations for ZO-1 and VE-cadherin (Fig. 5b, d). Maximal continuous claudin was found to be approximately 35% with 1d cAMP, higher than the approximate 30% observed with 1d cAMP treatment during 4-day culture. Punctate claudin, however, was unchanged with cAMP treatment and was presented at comparable levels to those found during 4-day culture. With cAMP treatment, perpendicular claudin was found at similar levels between 4-day and 7-day culture, though 6d cAMP treatment significantly decreased presentation to the approximate levels of 4-day culture with 0d cAMP.

### Transwell permeability (and TEER) assays are insufficient for correlative assessment of junction phenotype and barrier properties

Typically, continuous, linear adherens junctions are thought to be indicative of stable, mature EC barriers, while immature junctions are presented as punctate or perpendicular regions of protein [28]. As such, discontinuous junctions are typically linked with decreased barrier function, such as increased permeability [37]. Since the conditions tested here generated varied presentations of continuous and discontinuous junctions, we aimed to use these conditions to probe the influence of junction phenotype on barrier integrity using traditional measurement techniques (e.g., Transwell assays).

**Fig. 6:**
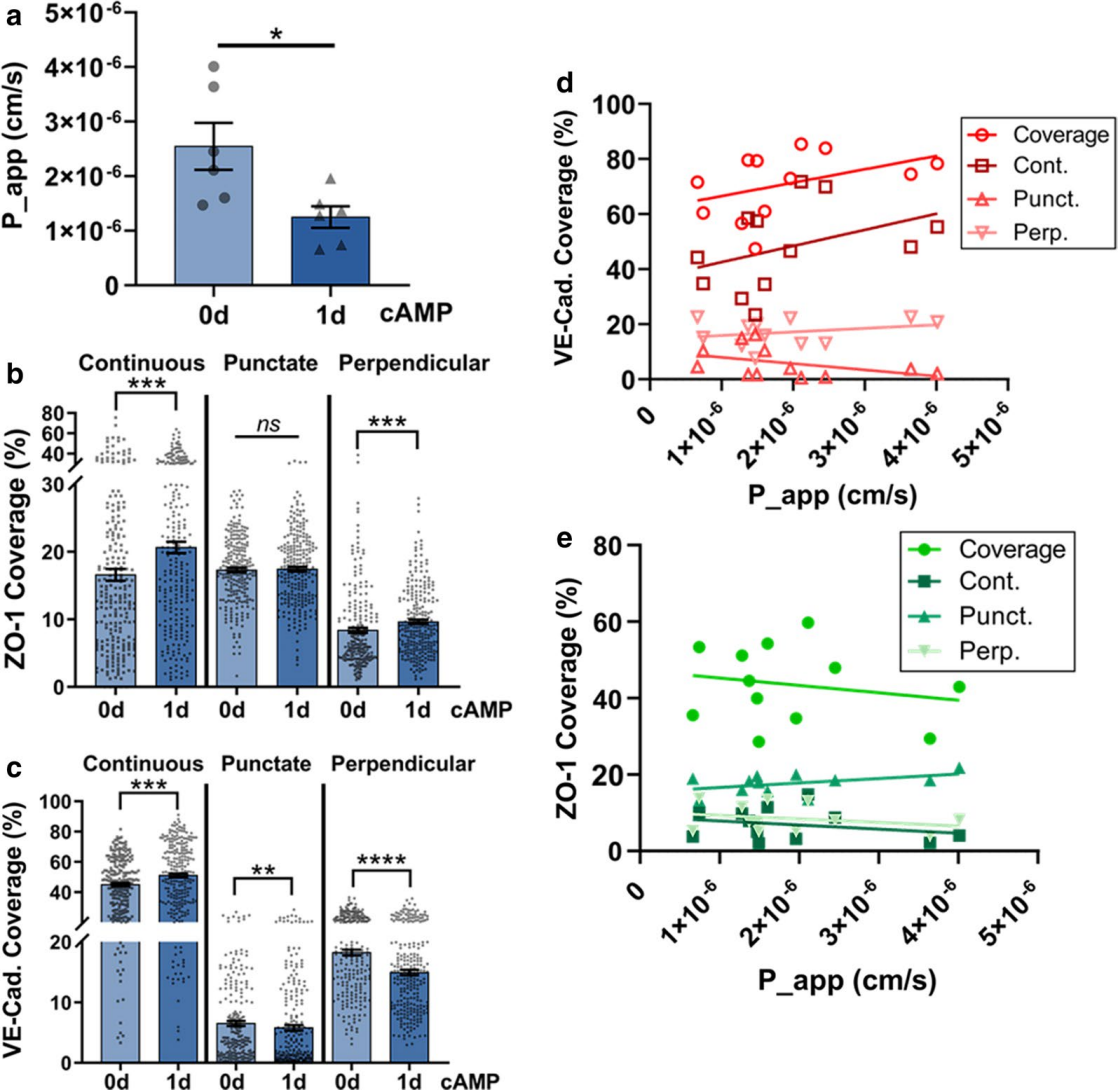
Transwell permeability assay. **a** Apparent permeability coefficient (P_app) of HBMECs cultured for 2 days with 0d or 1d cAMP treatment. N = 6, where N is the number of inserts measured over 3 trials. Inserts were analyzed using the JAnaP for ZO-1 (**b**) and VE-cadherin (**c**), and the correlation between junction coverage and P_app were evaluated (**d**, **e**). A linear regression rendered the slope of all relationships non-significantly non-zero. For (**b**, **c**), 232 ≤ N ≤ 244, where N is the number of cells, and for (**d**, **e**), N = 12, where N is the number of inserts pooled between the 0d and 1d cAMP conditions. The Mann–Whitney test was used to calculate significant differences for each parameter, where ns = p > 0.05, *p < 0.05, **p < 0.01, ***p < 0.001 and ****p < 0.0001

First, we performed a permeability assay by culturing HBMECs for 2 days on FBN-coated Transwell inserts, comparing the effects of 0d and 1d cAMP treatment on the permeability of 70 kDa FITC-dextran (Fig. 6). Since we observed a significant increase in junction coverage with 1d cAMP treatment in 2-day culture above, we expected to see decreased permeability with cAMP supplements. Indeed, the apparent permeability coefficient (P_app) decreased with 1d cAMP (Fig. 6a). To correlate these permeability values with junction presentation, the inserts were imaged and analyzed using the JAnaP (Fig. 6b, c), and the P_app values for each sample were plotted against calculated junction coverage values (Fig. 6d, e). Surprisingly, no significant correlation was found between junction coverage and permeability, which could suggest that ZO-1 and VE-cadherin phenotype have only a limited influence on the global permeability of the monolayer to this FITC-dextran molecule, if at all. This result is very unlikely given the plethora of literary evidence suggesting otherwise [38–42]. Importantly, however, many of these reports are qualitatively correlative between immunostaining and permeability measurement, and not a quantitative correlation between permeability and junction presentation. Based on our results above in Figs. 2 and 6a, we could draw a similar conclusion that increased continuous junctions is linked with decreased permeability. This conclusion, however, assumes that the junction presentation of the cells within the two different experimental setups is consistent. To probe the validity of this assumption, we investigated the shape and junction characteristics of cells cultured on Transwell inserts (Fig. 6, Additional file 1: Figure S11) to compare them against the results when cells were cultured on glass bottom plates (Fig. 2, Additional file 1: Figure S3). On glass, 1d cAMP treatment led to a decrease in cell area from approximately 2000 μm^2^ to 1500 μm^2^. Cells cultured on the inserts, however, were approximately 1700 μm^2^ in area regardless of cAMP treatment (Additional file 1: Figure S11). In both cases, circularity and solidity were similar with and without cAMP. While cAMP treatment increased continuous junctions in both cases, the resultant increase in overall coverage of continuous junctions was less on the inserts compared to glass. Specifically, continuous ZO-1 and VE-cadherin, respectively reached approximately 20% and 51% on the inserts, compared to the approximate 32% and 66% coverage values observed on glass.

We also measured TEER to probe barrier integrity and correlate the results with JAnaP analysis to gain insight into barrier resistance as a function of junction phenotype (Additional file 1: Additional Method S1). The 4-day experiment (Fig. 3b) on FBN with 0d, 1d, and 3d cAMP treatment was selected to provide increased variation of the total junction coverage, expecting increased TEER with increased cAMP treatment (Additional file 1: Figure S12). Surprisingly, changes in TEER with cAMP were only minor and non-significant. Similar to the results from the permeability study, no correlation between junction presentation and resistance measurements were observed. Again, both cell area and junction presentation differed on the Transwell inserts (Additional file 1: Figure S13) compared to culture on glass (Fig. 3 and Additional file 1: Figure S6) and did not respond to cAMP treatment in the same manner. While continuous ZO-1 on FBN-coated glass increased from approximately 10% with 0d cAMP, to 20% with 1d cAMP, and further to 40% with 3d cAMP (Fig. 3), the presentation on FBN-coated inserts increased from approximately 20% with 0d cAMP to approximately 35% with 1d and 3d cAMP (Additional file 1: Figure S13). Similarly, while continuous VE-cadherin increased from approximately 15% with 0d cAMP, to 45% with 1d cAMP, and further to 60% with 3d cAMP on FBN-coated glass, the presentation on FBN-coated inserts increased from approximately 50% with 0d cAMP treatment to approximately 58% with 1d and 3d cAMP. For both ZO-1 and VE-cadherin, this result suggests that without cAMP supplements, Transwell inserts promote enhanced continuous junction presentation compared to culture on glass.

Overall, this motivated the use of a more localized assay to understand the effects of junction phenotype on local barrier function. Specifically, the ability to correlate local junction presentation with local barrier properties in situ was needed to mechanistically quantify junction phenotype in conjunction with permeability.

### Local permeability assay reveals correlation between discontinuous junctions and barrier penetration

**Fig. 7:**
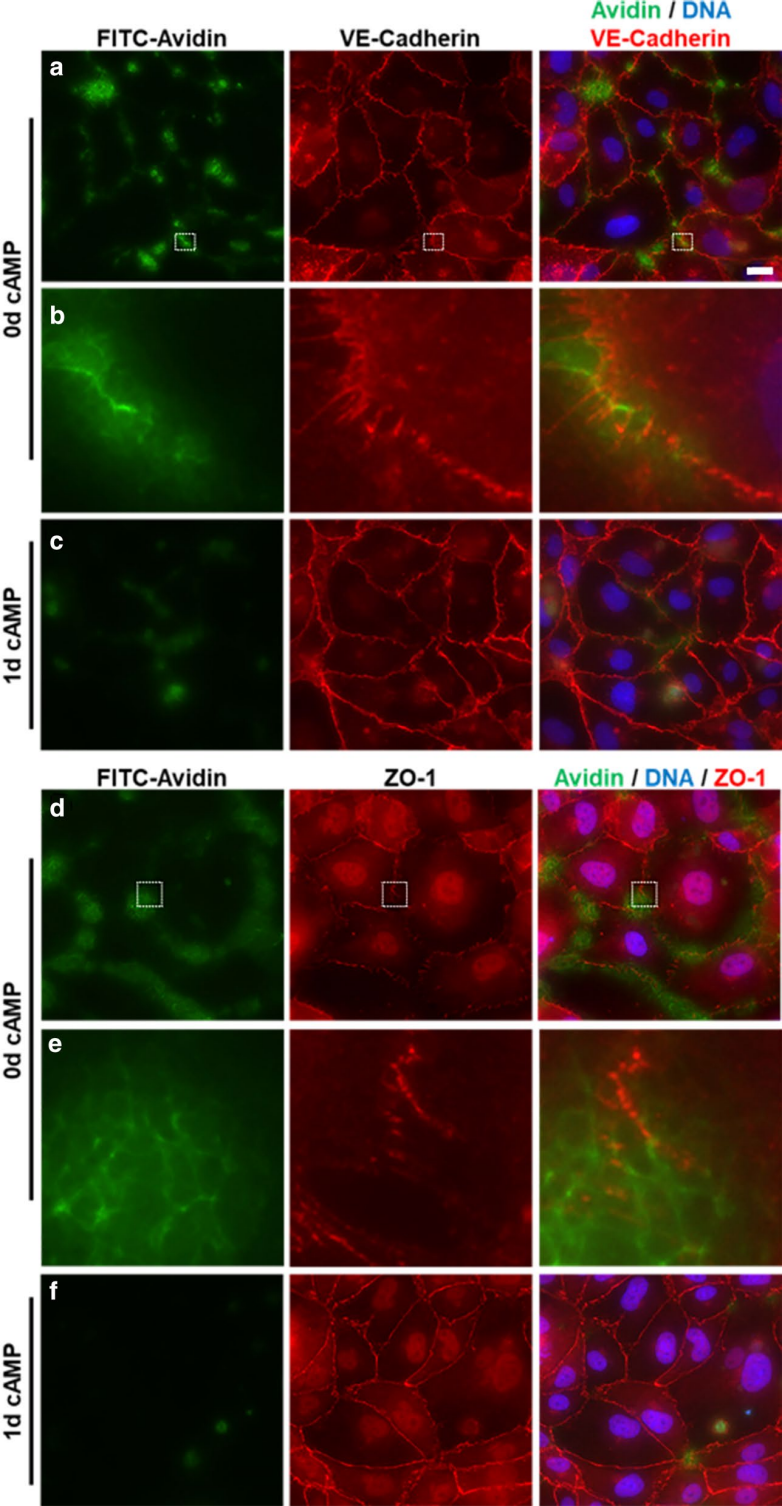
Local permeability assay. Immunofluorescence images of HBMECs cultured for 2 days on b-FBN with 0d of 1d cAMP, treated with FITC-avidin (green), then stained for VE-cadherin (row A, red), ZO-1 (row C, red), and DNA (blue). Rows B and C provide a zoomed-in view of the region outlined in the white-dotted box in the respective images (scale bar = 20 μm, applies to rows A and C)

To circumvent the challenges faced in the Transwell permeability and TEER assays, we adapted the XPerT assay [35] to detect regions of local monolayer permeability in situ. This technique enables visualization of barrier permeation via FITC-avidin-binding to biotinylated-FBN (B-FBN), in parallel with junction immunostaining. Here, we used this assay in conjunction with the JAnaP to quantitatively study junction phenotype and site-specific barrier permeability. Figure 7 presents representative images of VE-cadherin (A–C) and ZO-1 (D–F) in HBMECs cultured for 2 days. Note that no change in cell area was observed between FBN and B-FBN substrates, suggesting that the biotin label does not significantly affect HBMEC adhesion or spreading to the matrix (Additional file 1: Figure S14A). As expected, significantly more FITC-avidin was found to penetrate the endothelial cell barrier under conditions with 0d cAMP as compared to 1d cAMP treatment (Additional file 1: Figure S14B).

**Fig. 8:**
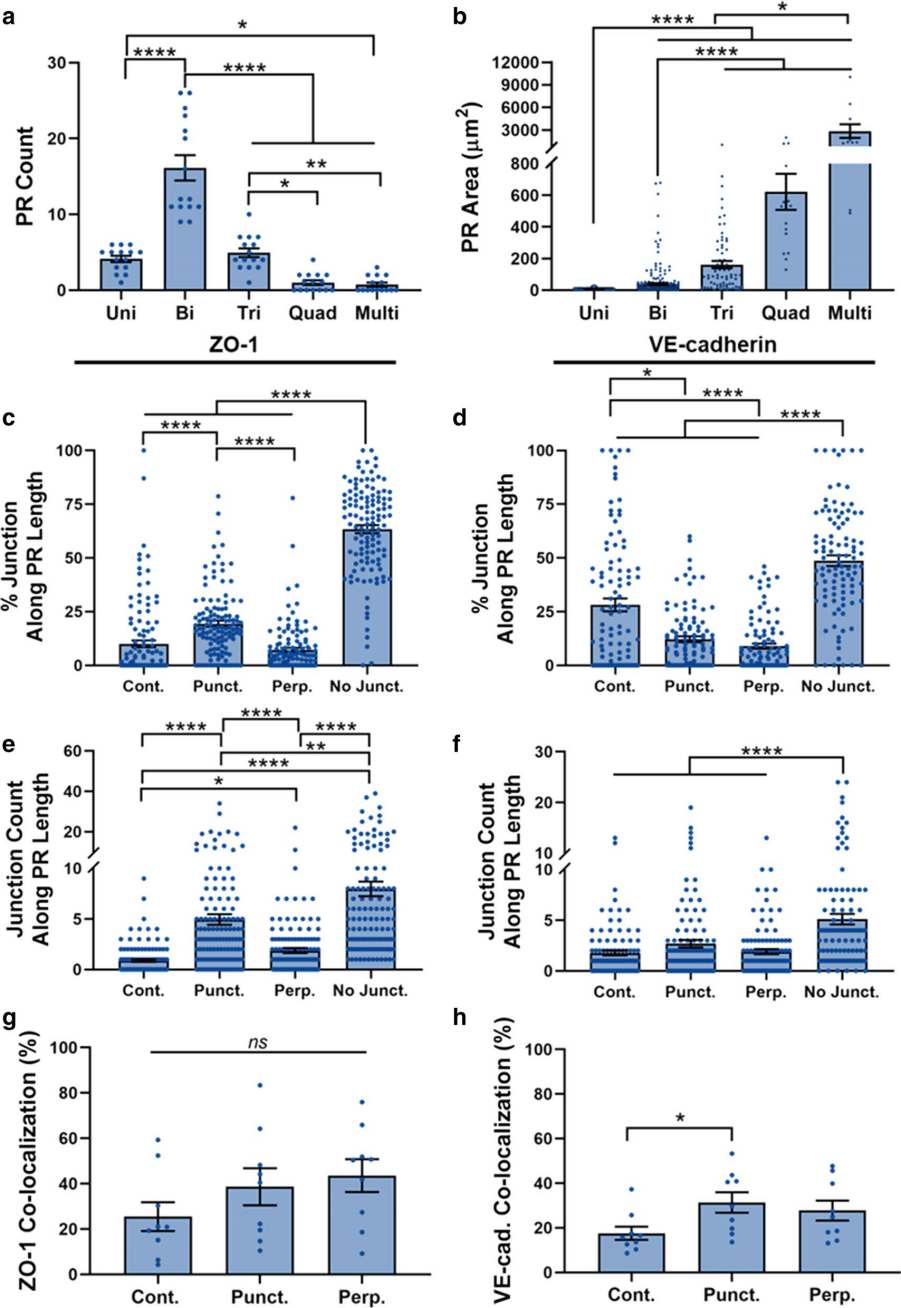
Permeated region and junction presentation analysis. The average number of each PR type per image is presented in **a** while the average size of each PR type is presented in **b**. N = 15 for (**a**) where N is the number of images. 11 ≤ N ≤ 247 for (**b**) where N is the number of PRs. Percentage (**c**, **d**) and count (**e**, **f**) of ZO-1 (left column) and VE-cadherin (right column) junctions along the cell edges co-localized with PRs. N = 105 for VE-cadherin and 126 for ZO-1, where N is the number of PRs. The co-localization of ZO-1 (**g**) and VE-cadherin (**h**) with PRs. N = 9, where N is the number of images. The Kruskal–Wallis test with a Dunn’s multiple comparison test was used to calculate significant differences for **a**–**f** and a Mann–Whitney test was used for (**g**, **h**), where ns = p > 0.05, *p < 0.05, **p < 0.01, and ****p < 0.0001

To start, we characterized the permeated regions (PR) of the monolayers, since the number of cells corresponding to PRs was not always consistent. We therefore categorized each PR based on the number of cells with which it was associated (i.e., Uni, Bi, Tri, Quad, or Multi). To quantify each of these instances, we averaged the number of times each category was present within each image (Fig. 8a). Note that these results were calculated using the monolayers immunostained for VE-cadherin, though similar results were observed when calculated for the ZO-1-stained images (Additional file 1: Figure S15). Bi-cellular PRs were the most consistent PR, with about 16 PRs per image (Fig. 8a). Larger PRs such as Quad or Multi were much less common, occurring less than or equal to one time per image. Size analysis indicated that PR area significantly increased with each additional cell contact, such that Uni PRs were smallest and Multi PRs were much larger (Fig. 8b).

**Fig. 9:**
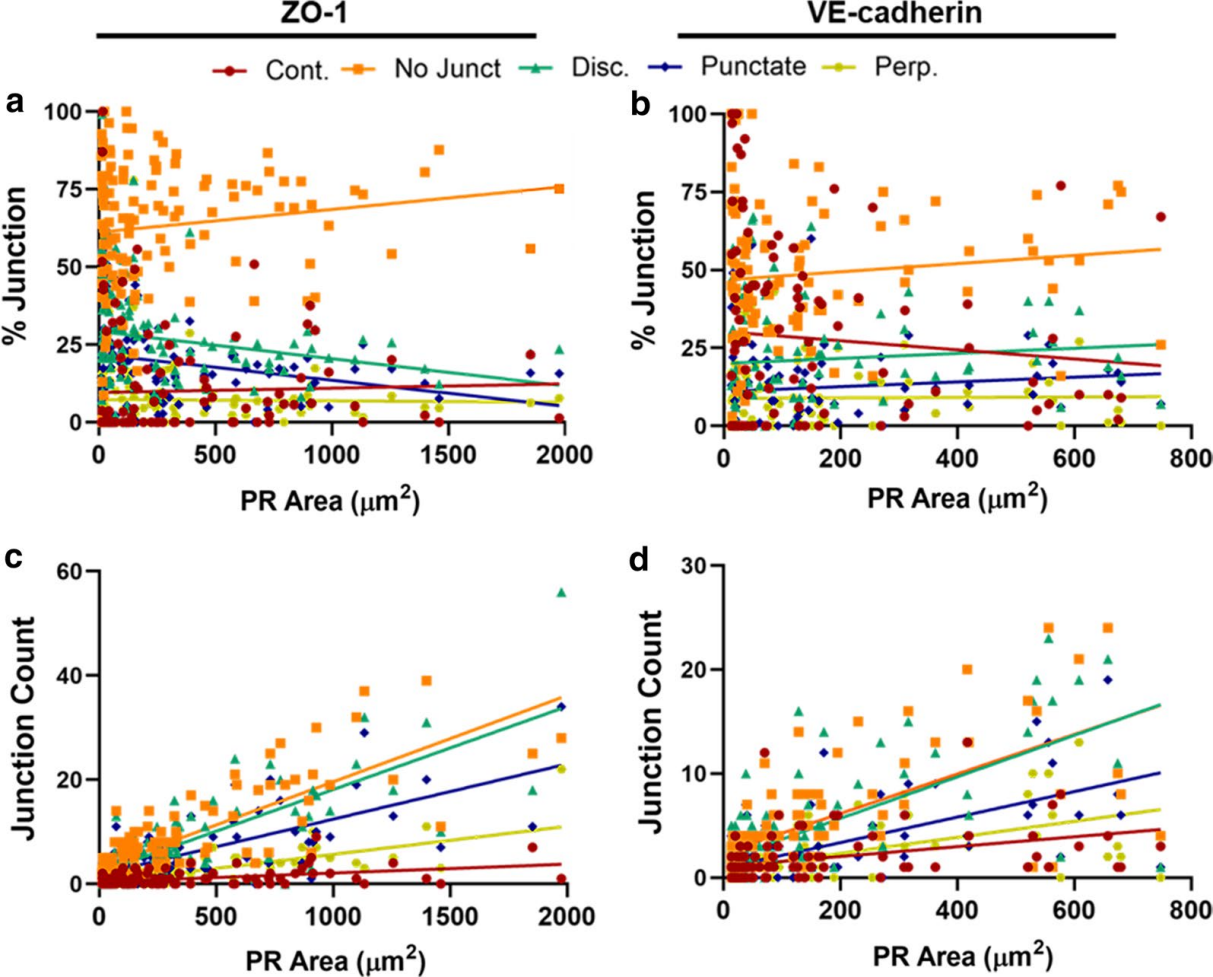
Junction Presentation versus PR Area. The correlation between PR area and the percent (**a**, **b**) and count (C,D) each continuous (Cont.), discontinuous (Disc.), and no junction (No Junct.) regions at the cell edge that are co-localized with a PR for ZO-1 and VE-cadherin. All results were fit using a linear regression. N = 105 for (**a**, **c**) and 124 (**b**, **d**), where N is the number of PRs. The Cont., No. Junct. Disc., Punctate, and Perp. R^2^ values are as follows: 0.001, 0.019, 0.039, 0.044, and 2.11E−4 for (**a**), 0.008, 0.009, 0.007, 0.010, and 9.80E−5 for (**b**) 0.213, 0.662, 0.617, 0.510, and 0.546 for (**c**), 0.143, 0.451, 0.493, 0.387, and 0.310 for (**d**). See Additional file 1: Table S4 for a summary of the statistical analysis for slope significance

Next, we investigated the types of junctions present at the PRs. We found the regions of cell path overlapping PRs were dominated by no junction regions for both VE-cadherin and ZO-1, though punctate junctions were also increased for ZO-1 (Fig. 8c–f). This suggests that in regions where FITC-avidin penetrated the barrier, the cell edge was most commonly covered by regions of “no junction”, and also significantly covered by punctate ZO-1. To gauge how frequently each junction type corresponded with a PR, we calculated the co-localization percentage for each image. While co-localization was greater for punctate VE-cadherin versus continuous junctions, no difference in co-localization amongst ZO-1 junctions was observed (Fig. 9g, h). Interestingly, the co-localization for both VE-cadherin and ZO-1, ranging from about 18–44% for all junctions, suggests that while punctate junctions are more likely to associate with a PR, their presence does not necessarily indicate a permeable region of the monolayer.

We were then curious to see if the amount of any one junction type (or no junction region) would instead correlate with “how permeable” the permeable regions were. We measured the extent of permeability as the area of the PR and investigated the correlation between PR area and junction presentation (Fig. 9). Note that these graphs have excluded 2 very large Multi PRs that were likely affected by more than just the local junction presentation of these proteins. Since the percentage of no junction regions dominated the PR length for both VE-cadherin and ZO-1, the magnitudes of values on this line were greater than either continuous or discontinuous (Fig. 9a, b). For VE-cadherin, there was no statistically significant correlation between percent junction (of any type) and PR area (Additional file 1: Table S7). For ZO-1, however, discontinuous junctions showed a significant correlation (p < 0.05, R^2^ = 0.039). This was likely driven by punctate junctions (p < 0.05, R^2^ = 0.044), since a significant correlation was found for punctate junctions but not perpendicular junctions. On the other hand, analysis of the junction counts showed significant trends for every condition. Since the junctions are inherently categorized by size (i.e., continuous are at least 15 pixels (or ~ 2.7 μm) long), looking at the number of junctions considers the smaller sizes of discontinuous junctions relative to continuous junctions, and that could be inadvertently skewing the percentage results. For both VE-cadherin and ZO-1, all junction types presented a significant correlation (p < 0.0001), with discontinuous and no junctions showing a greater positive correlation compared with continuous junctions (Fig. 9 and Additional file 1: Table S7). Together this suggests that size of the PR, or how permeable the barrier is as permeable regions, is equally influenced by the presentation of discontinuous junction and no junction, with continuous junctions playing less of a role.

## Discussion

While the influence of junction protein localization and presentation at the cell–cell borders of ECs on barrier properties has been previously investigated, these studies have been performed in a primarily qualitative manner and have lacked the quantification of junction phenotype. Development of the JAnaP has enabled the quantitative analysis of cell–cell junctions in situ, thereby permitting the study of junction phenotype on EC barrier properties in a calculated manner. Here, we varied cell culture parameters to understand their influence on junction presentation, and then used them to probe the effects on barrier permeability.

Despite the different properties of the six difference matrices studied here, [13, 43] use of different substrate coatings had almost no effect on cell shape factors and had the strongest influence on continuous (and in some cases, punctate) ZO-1 and VE-cadherin junctions. CN, Fbn, F:C:L, and HA/Gtn, all induced similar levels of total junction coverage, in line with previous reports for various brain EC types [14, 15, 44], while CIV and LN induced less junction localization in some cases. The result that LN induced a less optimal BBB phenotype was not surprising, since previous reports with iPSC-derived brain ECs reported the lowest TEER values and occludin expression on LN compared to other proteins, including several that were studied here [13]. That study also reported the greatest TEER values on FBN, supporting our result that, while marginal, FBN induced the greatest junction protein coverage [13]. Others have also reported the importance of FBN in brain endothelial cell culture to initiate and maintain a BBB phenotype. For example, Tilling et al. have suggested that FBN influences porcine brain capillary endothelial cell differentiation [45], and others have reported high resistance values in these cells only in the presence of astrocyte- and pericyte-derived matrices, which were found to contain large amounts of FBN (relative to CIV) [46, 47]. Interestingly, while increased claudin expression was found on matrices with increased FBN, ZO-1 expression was consistent regardless of FBN or CIV composition, in line with our results for ZO-1 on these matrices at longer culture times. The addition of cAMP supplements was found to have the greatest effect in junction presentation, increasing continuous junctions in almost every case. This was not surprising given the significant evidence that these supplements improve the barrier phenotype in ECs [24, 25]. Of specific relevance, one study reported increased TEER and localization of ZO-1 and VE-cadherin, showing a more linear morphology (assessed qualitatively) in HUVECs treated with the same concentrations of cAMP supplements for 1 day, supporting our results in this study [31]. Interestingly, increasing culture time in the presence of cAMP did not increase junction coverage for ZO-1 or VE-cadherin, and required longer cAMP treatment to reach similar presentation values observed in shorter experiments (FBN results presented in Additional file 1: Figure S16). This was surprising, since barrier maturity is thought to correlate with a more continuous, linear junction phenotype, but here required additional biochemical signaling. Furthermore, it seemed that time of culture prior to adding cAMP affected junction organization after cAMP was added. Future work should explore the time-dependent mechanisms driving cell–cell junction formation and maturation. Notably, a total culture time of 2-days or 7-days generally increased continuous and perpendicular junctions compared to 4-day experiments for both 0d and 1d cAMP treatment groups, suggesting total culture time does influence junction presentation but this effect seems to be mitigated with longer cAMP treatment. This could possibly involve cell contractility pathways, since cAMP reportedly inhibits Rho/ROCK signaling in endothelial cells, which blocks myosin light chain phosphorylation [48], leading to increased linear VE-cadherin formation [49], and presumably, decreased cell contractility. This contractility pathway may be downregulated during extended culture times, as the monolayer matures, and cells secrete new extracellular matrix. Then, addition of cAMP may have less of an effect because the Rho/ROCK pathway has already been downregulated. Future work could indeed explore this hypothesis.

For claudin-5, on the other hand, we did observe increased presentation with increased culture time, since no claudin was observed at 2 days and a maximal value was observed with 1d cAMP treatment during 7-day culture. This effect of culture time was expected, since tight junctions form after adherens junctions, requiring adherens junction structure for tight junction organization [2]. This difference in response of claudin to cAMP treatment time compared to VE-cadherin and ZO-1 was interesting, and possibly indicates different mechanisms regulating tight and adherens junction proteins. The requirement for increased cAMP signaling with increased culture time for ZO-1 and VE-cadherin did reach a limit, however, since 6d cAMP treatment during 7-day culture led to a decrease in junction architecture. This supports reports by Perrot et al., that prolonged activation of cAMP signaling can disrupt EC barriers [50]. Since cAMP is a regulator of gene expression, increases in its signaling can cause a delayed repression of Ras-related protein (R-Ras), which stabilizes VE-cadherin, thereby compromising junction stability and barrier integrity. Therefore, it is important to limit prolonged cAMP treatment and activation to maintain increased barrier function.

To correlate barrier properties with junction presentation, we performed Transwell permeability and TEER measurements. As expected, our permeability coefficient decreased with cAMP treatment, to a similar value reported for the B.end3 brain EC cell line in comparable conditions with 70 kDa Dextran (approximately 1E − 06 cm/s) [51]. The TEER measurements were on par with literature values, on the order of 10–30 Ω cm^2^ for static monoculture of B.end3 cells, [51] primary rat brain ECs, [52] purified murine brain ECs, [53] as well as for HBMECs [32]. The resultant trends, however, were not expected, since no significant differences were observed with cAMP treatment. Furthermore, neither TEER nor permeability showed a correlation with junction coverage. While this was surprising, there are several reasons to explain this unexpected result. First, the HBMEC monolayer may not be homogenous throughout the entire insert. Any gaps or regions of heterogeneity could lead to increased permeability, significantly skewing the P_app result, or decreased resistance due to “shortcuts to current flow” [54], since these are “bulk” barrier measurements. Second, the observed permeability response may be due to use of FITC-Dextran as the permeability marker, since molecules with different properties (e.g., size, charge) can permeate the barrier differently [54]. Third, the cells could be behaving differently due to the experimental conditions of the Transwell assays compared to on the glass bottom plates. Our JAnaP results suggest that indeed, the HBMECs are different in size and changes in junction architecture in response to cAMP are different compared to the glass bottom conditions. Notably, without cAMP treatment, we found Transwell inserts to induce higher continuous junction presentation in comparison with glass. These different trends for cell area and junction presentation could be explained by differences in the assay (e.g., treatment with FITC-Dextran, or cAMP supplements to both the apical and basal side), differences in the surface characteristics (e.g., charge, matrix coating efficiency), or possibly the different mechanical environments (i.e., stiff glass versus softer membrane, which we and other have previously shown to influence barrier integrity [4, 37, 55]). This could potentially explain the lack of correlation observed between junction phenotype and permeability, since one possibility is that the magnitude of change of each junction type did not vary enough to influence these measurements. This would mean that significantly more (or less) junction presentation would be required to alter the overall permeability or resistance of the barrier. Notably, continuous junction coverage alone fluctuated about 20–30% between cAMP treatments, suggesting that more extreme coverage values such as less than 10% or greater than 60% might be required to affect the output measurements. Other reports that qualitatively associated changes in junction phenotype and localization with barrier measurement often use treatments such as inflammatory cytokines (e.g., TNF-α) which could be altering other cell features that drive the changes in TEER rather than just phenotypic changes in junction presentation [56, 57]. For example, TNF-α treatment has been shown to induce the formation of filopodia, membrane ruffles, actin stress fibers, and intercellular gaps in human umbilical vein ECs [58]. Often in literature, the immunofluorescent staining of cells is performed, the TEER or permeability measurement is performed separately, and the two results are then correlated to infer function. We have shown here that cells can respond differently in the different systems and as such, caution should be taken when comparing cellular responses in different environments. These results motivated the use of a local assay to measure site-specific permeability in a single system that enables direct correlative measures. Since ECs can sense and respond to their microenvironment, EC phenotypes and barrier permeability are known to display spatial heterogeneity, further supporting the use of local studies to gain mechanistic insights into EC function [2, 59]. Furthermore, a local permeability assay would be more advantageous to use in complex microsystems where TEER or permeability measurements are not feasible. However, in some cases, global assays still provide valuable understanding, since the assessment of whole barrier function is important in, for example, in vitro modeling for the study of drug delivery systems.

The local permeability studies showed that permeated regions were most affected by the number of punctate and no junction regions of coverage, which had a positive correlation with PR area. This result supports the generally accepted idea that continuous junctions are indicative of a more mature and stable barrier [60–62]. This study also implicates punctate (versus perpendicular) junctions in the regulation of monolayer permeability, though future investigation is needed to unveil the differences between these two discontinuous junction types. It is important to note that this assay is only measuring the permeability of FITC-avidin and as such, could return different results for the permeability of molecules of different sizes or charges; or for different cell types transmigrating across the barrier. Use of the JAnaP with other local permeability assays for different molecules [63] or live cell imaging for cell transmigration [5, 64–66] could, therefore, provide additional insights into the effects of junction phenotypes. This is especially of interest to further study the differences in function between punctate and perpendicular junctions. Also of note, is that these studies were performed in static culture, despite the evidence suggesting the significant influences of mechanical cues in EC function [55]. Therefore, performing these correlative local permeability and TEER (or impedance) studies in a system that enables the incorporation of biomimetic microenvironmental cues (e.g., shear stress [13], substrate stiffness [28]) to probe the interplay of these parameters on a local scale is an important future application.

Overall, this study highlights the capabilities of combining junction phenotyping and assessment of barrier function for the mechanistic study of the BBB, and possibly other EC and epithelial barriers. Together, our data *quantitatively* suggest that increased continuous junction presentation is associated with a less permeable barrier, with increased gaps or discontinuous junctions indicating increased permeability. Our methods could provide valuable quantitative insights into time-dependent changes in junction architecture that occur in different biochemical or mechanical conditions. Understanding what conditions influence junction presentations and how that affects barrier properties could lead to therapeutic development for diseases associated with BBB dysfunction or delivery mechanisms capable of traversing healthy barrier systems.

## Conclusion

In summary, we investigated the influence of cell culture parameters such as matrix protein coating, culture time, and cAMP treatment, and used the JAnaP to quantify their role in cell and junction morphology. While protein coating seemed to have only a modest effect on these parameters, cAMP treatment significantly increased continuous junction presentation. Total cell culture time did not increase junction presentation, but instead required increased cAMP treatment for protein coverage comparable to shorter culture time. No correlation between junction presentation and barrier permeability was found when comparing junction phenotype to Transwell-based TEER and permeability experiments, motivating the use of an assay that could instead capture cell-to-cell inhomogeneities rather than a “bulk” barrier measurement. A local permeability assay identified that barrier permeability most closely correlates with the number of gaps with no junction coverage, and by extension, the number of discontinuous junctions, present at the cell edge. Together this promotes the use of local measurement techniques to quantitatively study barrier function in conjunction with junction phenotype to investigate the mechanisms at play in functional and dysfunctional barrier systems.

## Supplementary information

**Additional file 1.** This Additional file contains Figures S1-S16, Tables S1-S7, and Additional Method S1.

## Data Availability

The JAnaP is available for download at https://github.com/StrokaLab/JAnaP.

## Abbreviations

BBB: Blood–brain barrier
B-FBN: Biotinylated fibronectin
CN: Collagen I
CPT-cAMP: 8-(4-Chlorophenylthio) adenosine-3′,5′-cyclic monophosphate sodium salt
CIV: Collagen IV
ECs: Endothelial cells
FBN: Fibronectin F:C:L—fibronectin + collagen IV + laminin
HBMEC: Human brain microvascular endothelial cell
HUVEC: Human umbilical vein endothelial cell
JANaP: Junction Analyzer Program
LN: Laminin
P_app: Apparent permeability coefficient
PBS: Dulbecco’s Phosphate-Buffered Saline containing calcium and magnesium
PR: Permeated region
RO-20-1724: 4-(3-Butoxy-4-methoxybenzyl) imidazolidin-2-one
TEER: Transendothelial electrical resistance
VE-cadherin: Vascular endothelial cadherin
ZO-1: Zonula occludens 1
