# Supplementary material for: Quantitatively relating brain endothelial cell–cell junction phenotype to global and local barrier properties under varied culture conditions via the Junction Analyzer Program
Source: Fluids Barriers CNS. 2020 Feb 11;17:16. doi: 10.1186/s12987-020-0177-y (PMC7014765; doi:10.1186/s12987-020-0177-y)
Supplement: Supplementary file 1 — Additional file 1. This Additional file contains Figures S1-S16, Tables S1-S7, and Additional Method S1. [file 12987_2020_177_MOESM1_ESM.pdf]

Additional File for:

Quantitatively relating brain endothelial cell-cell junction phenotype to global and local barrier properties under varied culture conditions via the Junction Analyzer Program

Kelsey M. Gray[1], Jae W. Jung[1], Collin T. Inglut[1], Huang-Chiao Huang[1,4], Kimberly M. Stroka[1,2,3,4#]

1) Fischell Department of Bioengineering, University of Maryland, College Park, MD 20742, USA

2) Biophysics Program, University of Maryland, College Park, MD 20742, USA

3) Center for Stem Cell Biology and Regenerative Medicine, University of Maryland – Baltimore, MD, 21201, USA

4) Marlene and Stewart Greenebaum Comprehensive Cancer Center, University of Maryland – Baltimore, MD 21201, USA

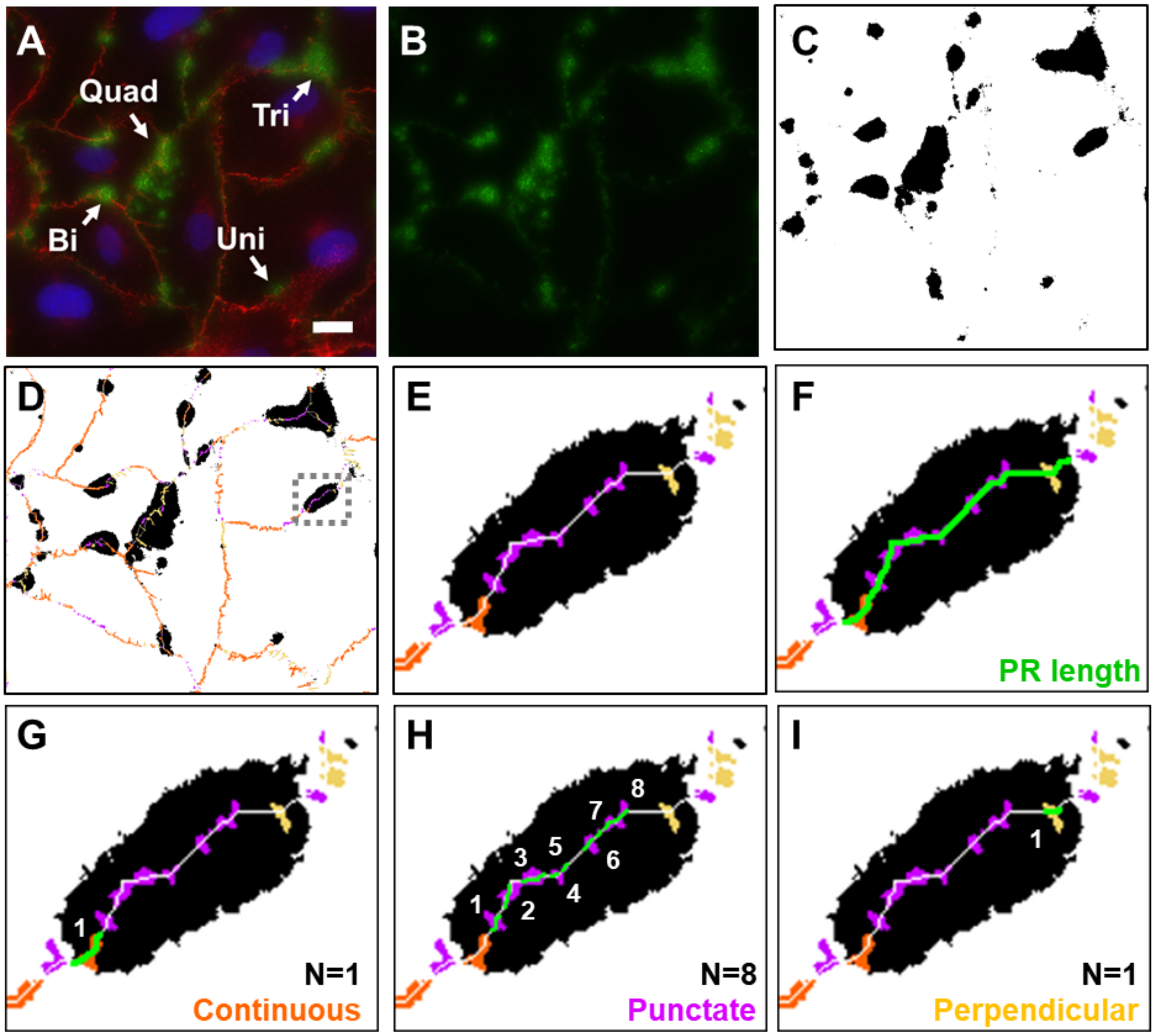

Figure S1. Local Permeability Analysis – Image Processing. Composite image of VE-cadherin (red) and FITC-avidin (green), labeled to identify examples of the PR categories (A). Images of bound FITC-avidin (B) are processed in ImageJ to generate 8-bit binary images of PRs (C). The raw junctional protein images are processed in the JAnaP to generate images of categorized junctions, which can be overlaid onto the PR images (D). Cropped images depicting the region in the gray-dotted box in (D) (E-I). Trace of the length of the cell edge that corresponds to the length of the PR (green) provides the PR length (F). Tracing the length of each junction type (green) provides the count and length of continuous (G), punctate (H), and perpendicular (I) junctions along the PR length. The numbers indicate a distinct junction piece. (scale bar = 20 µm, applies to A-D)

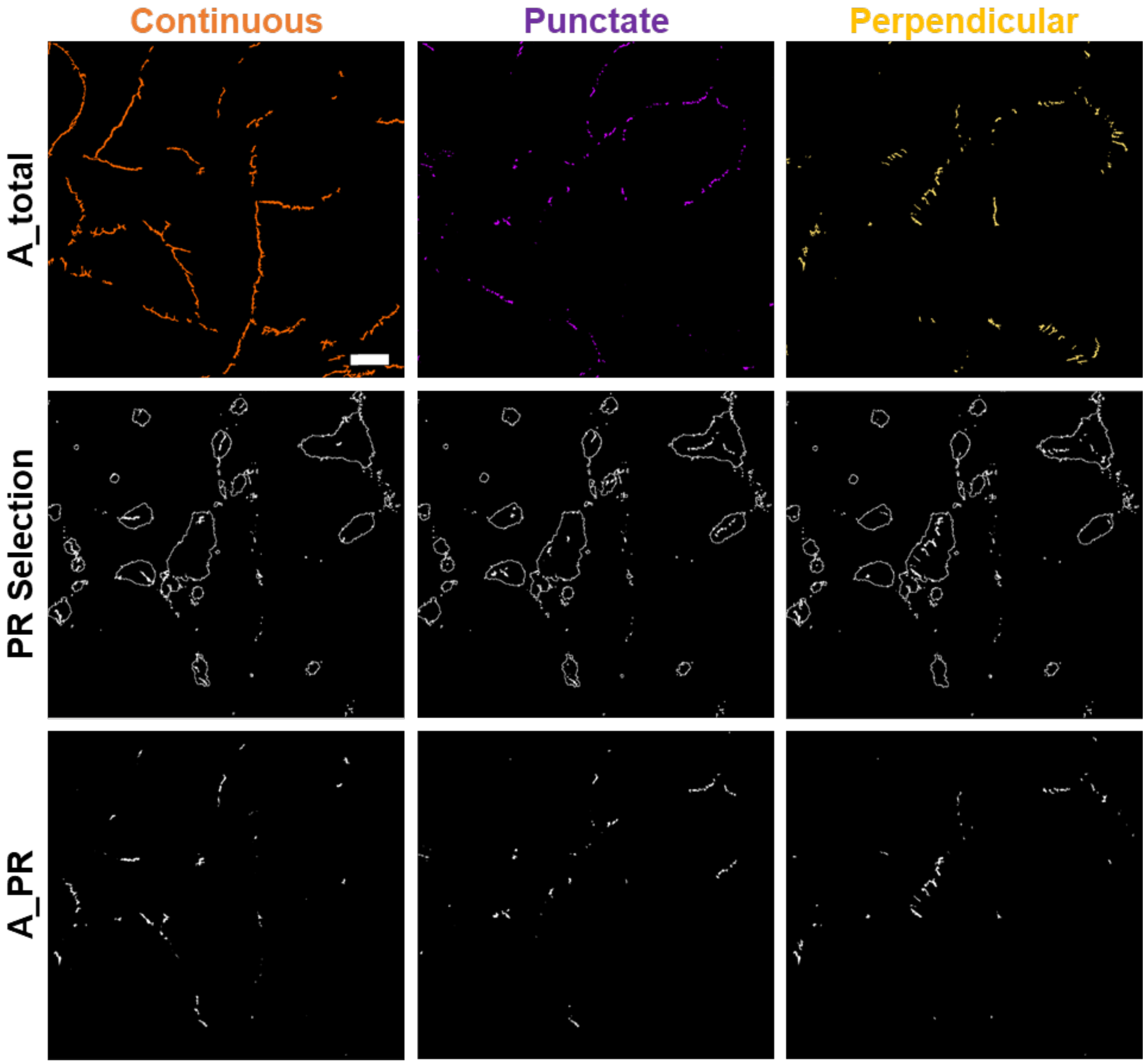

Figure S2. Local Permeability Analysis - Co-localization. Junctional protein images are processed in the JAnaP to categorize junctions, then each junction type was separated into a different image (top row). A selection in ImageJ was used to measure the total junction area (A_total) for each junction type within each image. A selection of the corresponding PR threshold image (Supplemental Figure S1.C) was then used to create a mask that was applied to each junction type (middle row) to remove all junctions that did not colocalize with a PR. Area measurement of remaining junctions (bottom row) provided the PR colocalized junction area (A_PR). The Co-localization (%) was calculated as (A_PR/A_total)*100.

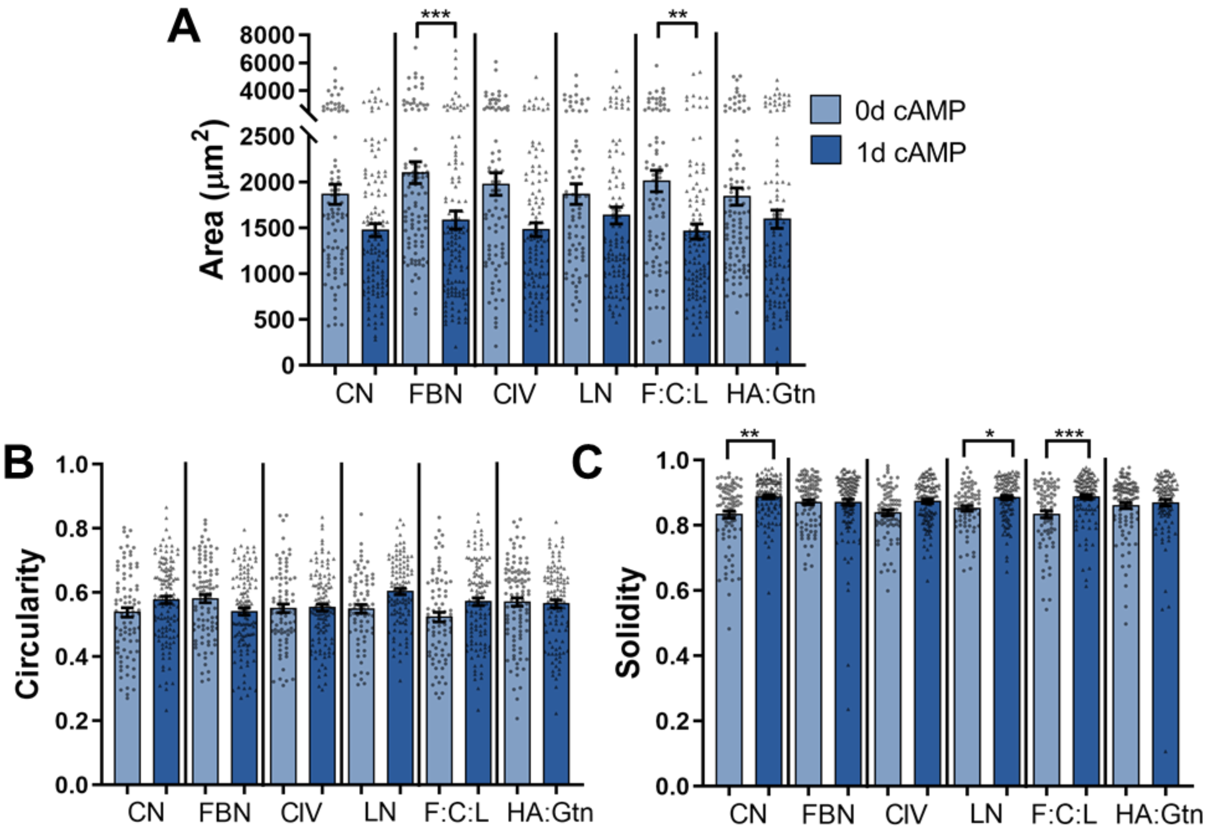

Figure S3. Cell Morphology Analysis for 2-day Culture. Cell area (A), circularity (B), and solidity (C) of HBMECs cultured on the 6 substrate coatings for 2 days, with and without 1d cAMP treatment. $72 \leq N \leq 125$, where N is the number of cells. The Kruskal-Wallis test was used to calculate significant differences, where * $p < 0.05$, ** $p < 0.01$, and *** $p < 0.001$. See Supplemental Table S1 for comparative statistical analysis between protein coatings.

Cell Area

Comparisons	0cAMP	1cAMP
CIV vs CN	ns	ns
CIV vs FBN	ns	ns
CIV vs FCL	ns	ns
CIV vs GG	ns	ns
CIV vs LN	ns	ns
CN vs FBN	ns	ns
CN vs FCL	ns	ns
CN vs GG	ns	ns
CN vs LN	ns	ns
FBN vs FCL	ns	ns
FBN vs GG	ns	ns
FBN vs LN	ns	ns
FCL vs GG	ns	ns
FCL vs LN	ns	ns
GG vs LN	ns	ns

Cell Circularity

Comparisons	0cAMP	1cAMP
CIV vs CN	ns	ns
CIV vs FBN	ns	ns
CIV vs FCL	ns	ns
CIV vs GG	ns	ns
CIV vs LN	ns	ns
CN vs FBN	ns	ns
CN vs FCL	ns	ns
CN vs GG	ns	ns
CN vs LN	ns	ns
FBN vs FCL	ns	ns
FBN vs GG	ns	ns
FBN vs **LN**	ns	**
FCL vs GG	ns	ns
FCL vs LN	ns	ns
GG vs LN	ns	ns

Cell Solidity

Comparisons	0cAMP	1cAMP
CIV vs CN	ns	ns
CIV vs FBN	ns	ns
CIV vs FCL	ns	ns
CIV vs GG	ns	ns
CIV vs LN	ns	ns
CN vs FBN	ns	ns
CN vs FCL	ns	ns
CN vs GG	ns	ns
CN vs LN	ns	ns
FBN vs FCL	ns	ns
FBN vs GG	ns	ns
FBN vs LN	ns	ns
FCL vs GG	ns	ns
FCL vs LN	ns	ns
GG vs LN	ns	ns

Table S1. Statistical Analysis for Cell Morphology of 2-day Culture. The comparison between each substrate protein with and without cAMP is presented, as calculated using the Kruskal-Wallis test with a Dunn's multiple comparison test. A red box marked with "ns" signifies $p > 0.05$. A green box signifies a significant difference, where ** $p < 0.01$ and bold text indicates which protein generated the higher value. Data corresponds to Supplemental Figure S3.

ZO-1 Coverage

Comparisons	0cAMP	1cAMP
CIV vs CN	ns	ns
CIV vs **FBN**	*	*
CIV vs FCL	ns	ns
CIV vs **GG**	**	ns
CIV vs LN	ns	ns
CN vs **FBN**	*	ns
CN vs FCL	ns	ns
CN vs **GG**	**	ns
**CN** vs LN	ns	*
FBN vs FCL	ns	ns
FBN vs GG	ns	ns
**FBN** vs LN	****	****
FCL vs GG	ns	ns
**FCL** vs LN	**	****
**GG** vs LN	****	****

ZO-1 Continuous

Comparisons	0cAMP	1cAMP
CIV vs CN	ns	ns
CIV vs **FBN**	*	*
CIV vs **FCL**	ns	*
CIV vs **GG**	**	**
CIV vs LN	ns	ns
CN vs FBN	ns	ns
CN vs FCL	ns	ns
CN vs GG	ns	ns
**CN** vs LN	ns	*
FBN vs FCL	ns	ns
FBN vs GG	ns	ns
**FBN** vs LN	**	****
FCL vs GG	ns	ns
**FCL** vs LN	*	****
**GG** vs LN	***	****

VE-Cad Coverage

Comparisons	0cAMP	1cAMP
CIV vs **CN**	**	ns
CIV vs **FBN**	**	ns
CIV vs FCL	ns	ns
CIV vs GG	ns	ns
CIV vs LN	ns	ns
CN vs FBN	ns	ns
CN vs FCL	ns	ns
CN vs GG	ns	ns
**CN** vs LN	***	ns
FBN vs FCL	ns	ns
FBN vs GG	ns	ns
**FBN** vs LN	ns	***
FCL vs GG	ns	ns
FCL vs LN	ns	ns
**GG** vs LN	*	ns

VE-Cad Continuous

Comparisons	0cAMP	1cAMP
CIV vs **CN**	**	ns
CIV vs **FBN**	ns	**
CIV vs FCL	ns	ns
CIV vs GG	ns	ns
CIV vs LN	ns	ns
CN vs FBN	ns	ns
CN vs FCL	ns	ns
CN vs GG	ns	ns
**CN** vs LN	*	ns
FBN vs FCL	ns	ns
FBN vs GG	ns	ns
**FBN** vs LN	ns	**
FCL vs GG	ns	ns
FCL vs LN	ns	ns
GG vs LN	ns	ns

ZO-1 Punctate

Comparisons	0cAMP	1cAMP
CIV vs CN	ns	ns
CIV vs FBN	ns	ns
**CIV** vs FCL	ns	*
**CIV** vs GG	ns	*
**CIV** vs LN	**	ns
CN vs FBN	ns	ns
CN vs FCL	ns	ns
CN vs **GG**	**	ns
CN vs LN	ns	ns
FBN vs FCL	ns	ns
FBN vs GG	ns	ns
**FBN** vs LN	****	ns
FCL vs GG	ns	ns
**FCL** vs LN	**	ns
GG vs **LN**	****	ns

ZO-1 Perpendicular

Comparisons	0cAMP	1cAMP
CIV vs CN	ns	ns
CIV vs FBN	ns	ns
CIV vs FCL	ns	ns
CIV vs GG	ns	ns
CIV vs LN	ns	ns
CN vs FBN	ns	ns
CN vs FCL	ns	ns
CN vs GG	ns	ns
CN vs LN	ns	ns
FBN vs FCL	ns	ns
FBN vs GG	ns	ns
**FBN** vs LN	***	ns
FCL vs GG	ns	ns
FCL vs LN	ns	ns
**GG** vs LN	**	ns

VE-Cad Punctate

Comparisons	0cAMP	1cAMP
CIV vs CN	ns	ns
**CIV** vs FBN	ns	**
CIV vs FCL	ns	ns
CIV vs GG	ns	ns
CIV vs LN	ns	ns
CN vs FBN	ns	ns
CN vs FCL	ns	ns
CN vs GG	ns	ns
CN vs LN	ns	ns
FBN vs FCL	ns	ns
FBN vs GG	ns	ns
FBN vs LN	ns	ns
FCL vs GG	ns	ns
FCL vs LN	ns	ns
GG vs LN	ns	ns

VE-Cad Perpendicular

Comparisons	0cAMP	1cAMP
CIV vs CN	ns	ns
CIV vs FBN	ns	ns
CIV vs FCL	ns	ns
CIV vs GG	ns	ns
CIV vs LN	ns	ns
CN vs FBN	ns	ns
CN vs FCL	ns	ns
CN vs GG	ns	ns
CN vs LN	ns	ns
FBN vs FCL	ns	ns
FBN vs GG	ns	ns
FBN vs LN	ns	ns
FCL vs GG	ns	ns
FCL vs LN	ns	ns
GG vs LN	ns	ns

Table S2. Statistical Analysis for Junction Phenotyping of 2-day Culture. The comparison between each substrate protein with and without cAMP is presented, as calculated using the Kruskal-Wallis test with a Dunn's multiple comparison test. A red box marked with "ns" signifies $p > 0.05$. A green box signifies a significant difference, where * $p < 0.05$, ** $p < 0.01$, *** $p < 0.001$, and **** $p < 0.0001$, and bold text indicates which protein generated higher coverage. Data corresponds to Figure 2.

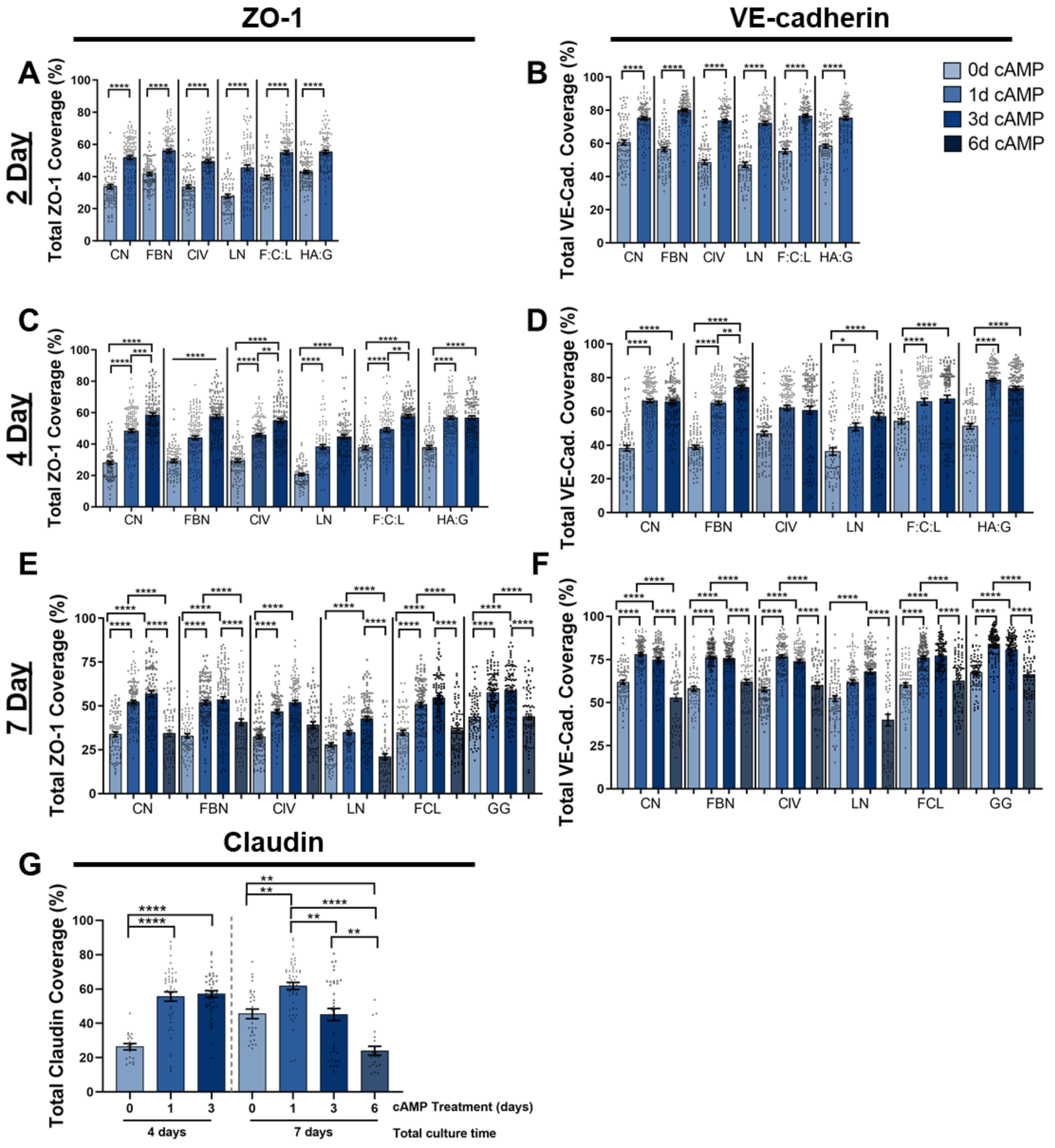

Figure S4. Total junction coverage. Edge presentation of ZO-1 (top left panels), VE-Cadherin (right panels), and Claudin-5 (bottom left) for 2-day (A-B), 4-day (C-D,G), and 7-day (E-G) experiments. Each junction type from Figures 2, S6, and S9 were summed to represent the total coverage of each junction protein. $72 \leq N \leq 125$ for (A-B), $77 \leq N \leq 145$ for (C-D), $56 \leq N \leq 126$ for (E-F), and $19 \leq N \leq 52$ for (G), where N is the number of cells. Statistical analysis was used to compare results within the same substrate protein group (A-F) and culture time (G). The Kruskal-Wallis test with a Dunn's multiple comparison test was used to calculate significant differences where * $p < 0.05$, ** $p < 0.01$, *** $p < 0.001$ and **** $p < 0.0001$.

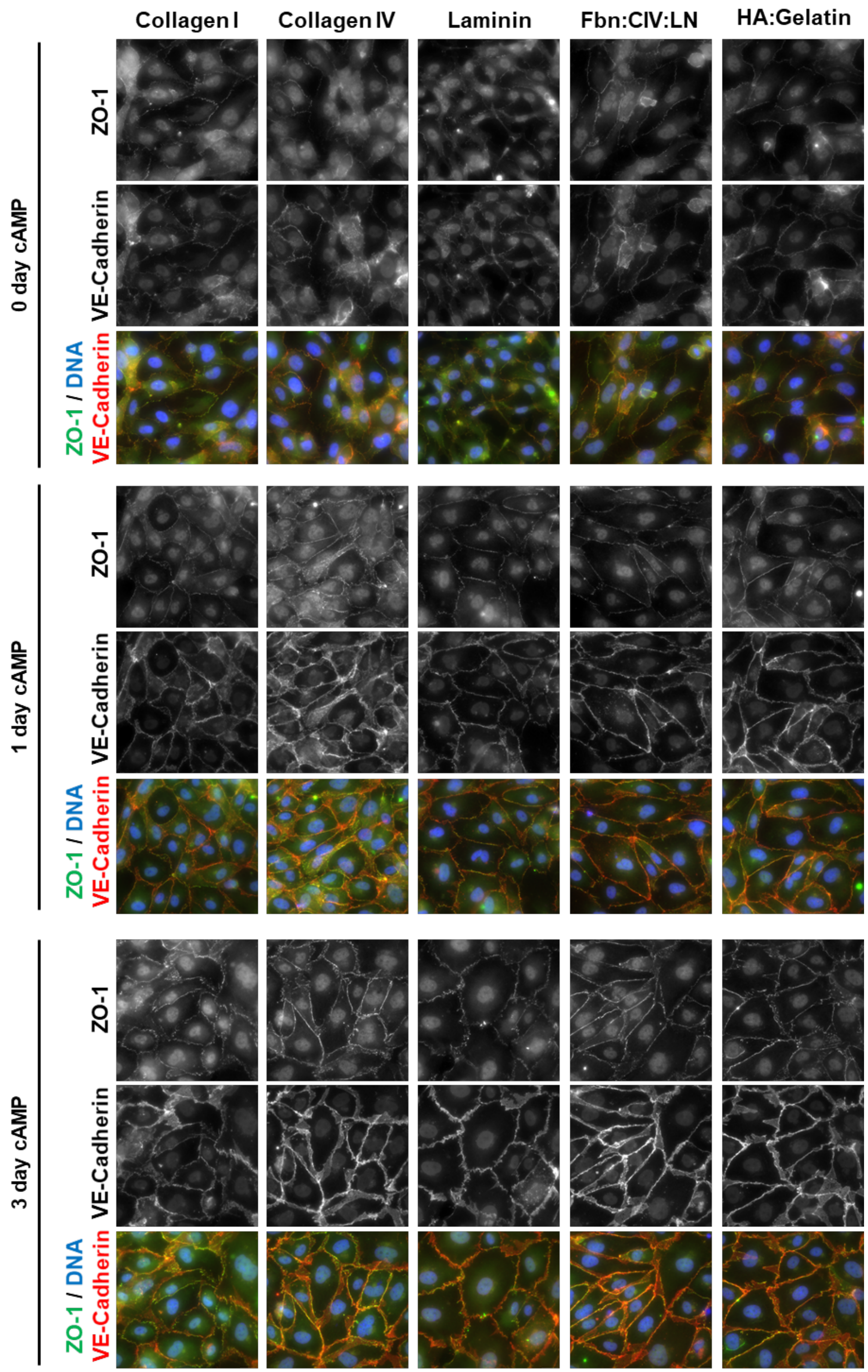

Figure S5. Immunofluorescence Images of HBMECs in 4-day Culture. HBMECs on 5 substrate coatings, cultured for 4 days with 0d, 1d, or 3d of cAMP treatment, stained for ZO-1 (green), VE-cadherin (red), and DNA (blue). (scale bar = 20 μm)

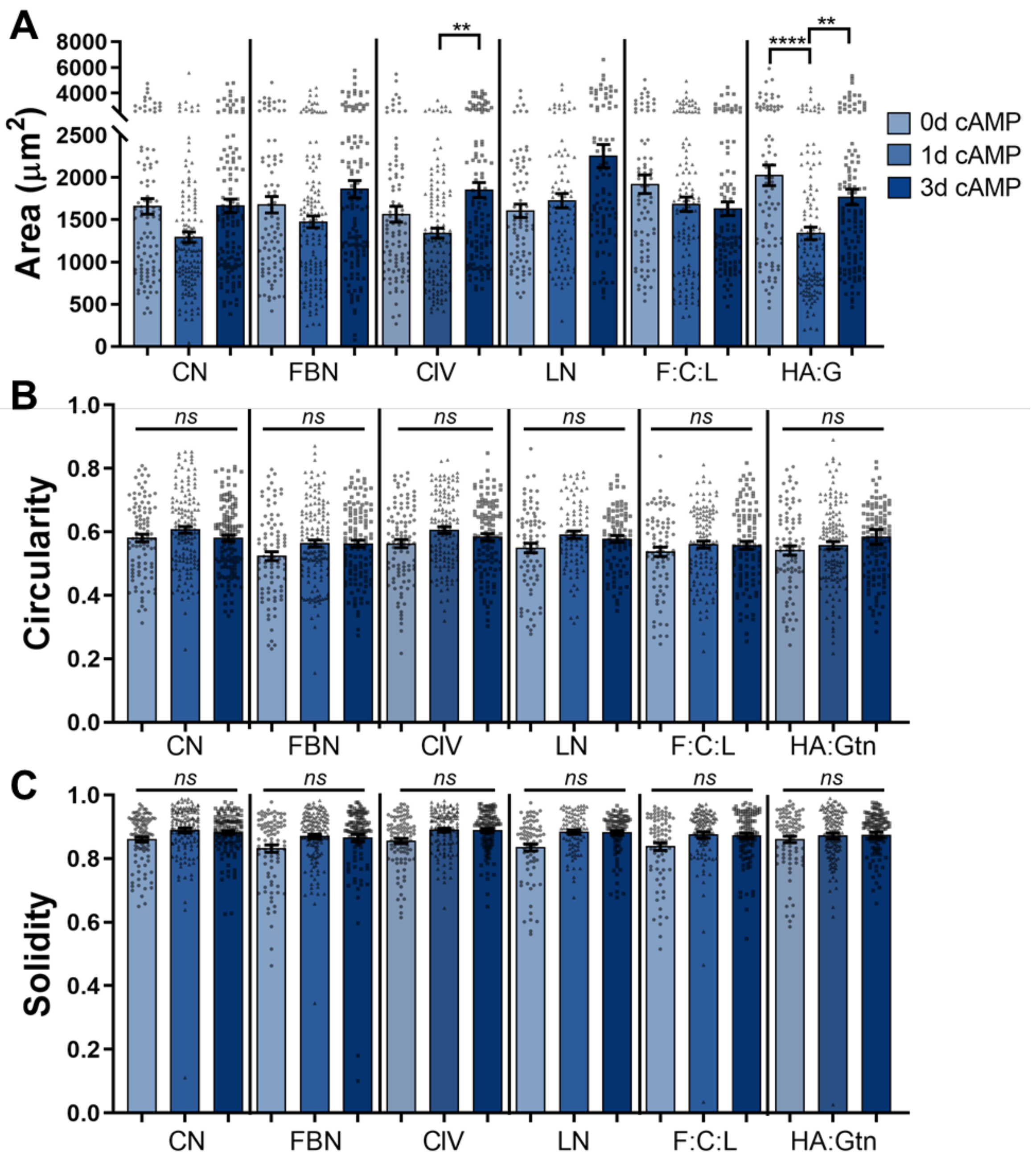

Figure S6. Cell Morphology Analysis for 4-day Culture. Cell area (A), circularity (B), and solidity (C) of HBMECs cultured on the 6 substrate coatings for 4 days, with 0d, 1d, or 3d of cAMP treatment. $72 \leq N \leq 125$, where N is the number of cells. The Kruskal-Wallis test was used to calculate significant differences, where ns = $p > 0.05$, ** $p < 0.01$, and **** $p < 0.0001$. See Supplemental Table S3 for comparative statistical analysis between protein coatings.

Cell Area

Comparisons	0cAMP	1cAMP	3cAMP
CIV vs CN	ns	ns	ns
CIV vs FBN	ns	ns	ns
CIV vs FCL	ns	ns	ns
CIV vs GG	ns	ns	ns
CIV vs LN	ns	ns	ns
CN vs FBN	ns	ns	ns
CN vs **FCL**	ns	*	ns
CN vs GG	ns	ns	ns
CN vs **LN**	ns	**	ns
FBN vs FCL	ns	ns	ns
FBN vs GG	ns	ns	ns
FBN vs LN	ns	ns	ns
FCL vs GG	ns	ns	ns
FCL vs LN	ns	ns	ns
GG vs **LN**	ns	**	ns

Cell Circularity

Comparisons	0cAMP	1cAMP	3cAMP
CIV vs CN	ns	ns	ns
CIV vs FBN	ns	ns	ns
CIV vs FCL	ns	ns	ns
CIV vs GG	ns	ns	ns
CIV vs LN	ns	ns	ns
CN vs FBN	ns	ns	ns
CN vs FCL	ns	ns	ns
CN vs GG	ns	ns	ns
CN vs LN	ns	ns	ns
FBN vs FCL	ns	ns	ns
FBN vs GG	ns	ns	ns
FBN vs LN	ns	ns	ns
FCL vs GG	ns	ns	ns
FCL vs LN	ns	ns	ns
GG vs LN	ns	ns	ns

Cell Solidity

Comparisons	0cAMP	1cAMP	3cAMP
CIV vs CN	ns	ns	ns
CIV vs FBN	ns	ns	ns
CIV vs FCL	ns	ns	ns
CIV vs GG	ns	ns	ns
CIV vs LN	ns	ns	ns
CN vs FBN	ns	ns	ns
CN vs FCL	ns	ns	ns
CN vs GG	ns	ns	ns
CN vs LN	ns	ns	ns
FBN vs FCL	ns	ns	ns
FBN vs GG	ns	ns	ns
FBN vs LN	ns	ns	ns
FCL vs GG	ns	ns	ns
FCL vs LN	ns	ns	ns
GG vs LN	ns	ns	ns

Table S3. Statistical Analysis for Cell Morphology of 4-day Culture. The comparison between each substrate protein with 0d, 1d, and 3d cAMP is presented, as calculated using the Kruskal-Wallis test with a Dunn's multiple comparison test. A red box marked with "ns" signifies $p > 0.05$. A green box signifies a significant difference, where * $p < 0.05$ and ** $p < 0.01$ and bold text indicates which protein generated the higher value. Data corresponds to Supplemental Figure S6.

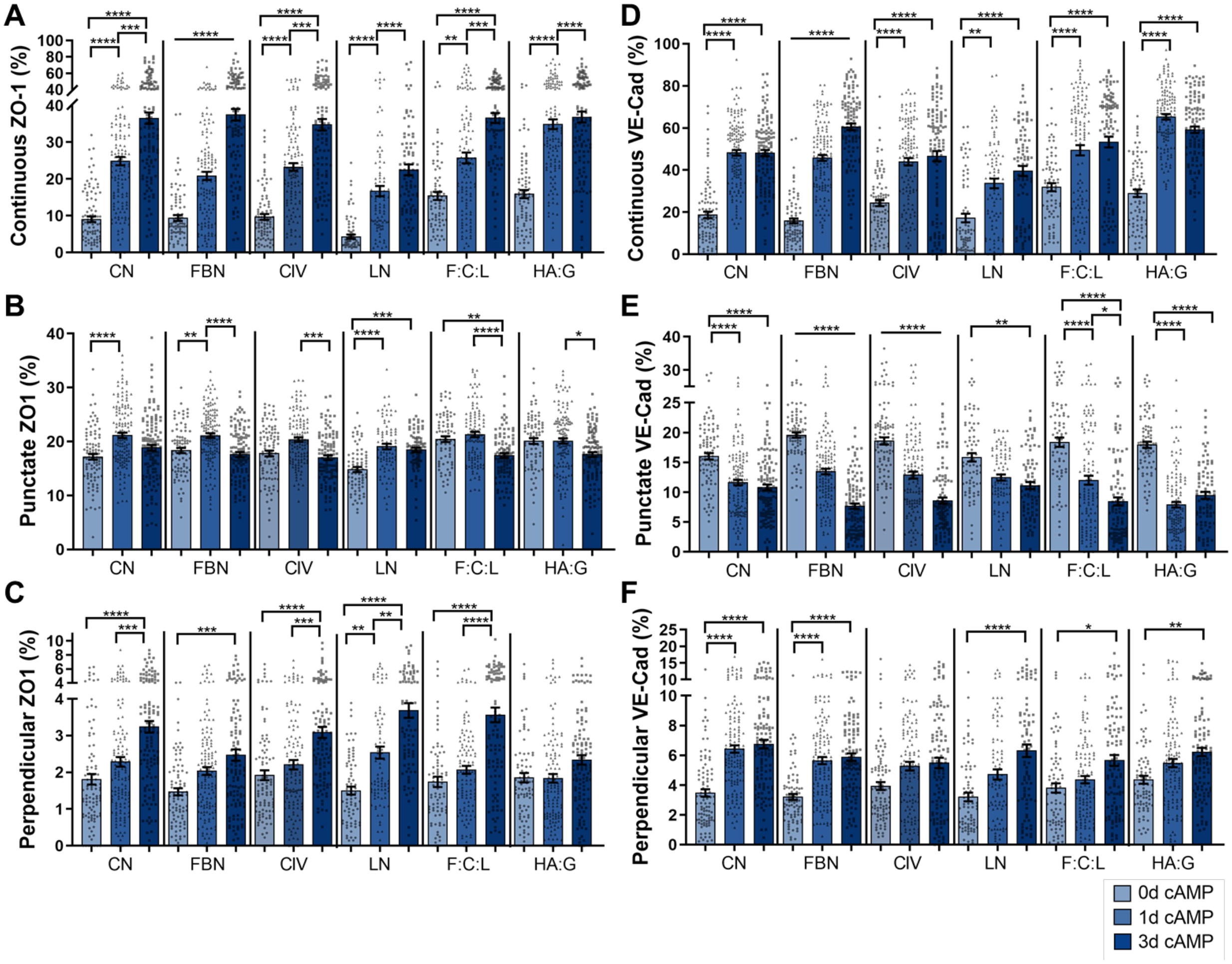

Figure S7. Junction Phenotype Analysis for 4-day Culture. Edge presentation of continuous (A), punctate (B), and perpendicular (C) junctions for ZO-1 (A-C) and VE-cadherin (D-F). $77 \leq N \leq 145$, where N is the number of cells. The Kruskal-Wallis test with a Dunn's multiple comparison test was used to calculate significant differences, where * $p < 0.05$, ** $p < 0.01$, *** $p < 0.001$, and **** $p < 0.0001$. See Supplemental Table S2 for comparative statistical analysis between protein coatings.

ZO-1 Coverage

Comparisons	0cAMP	1cAMP	3cAMP
CIV vs CN	ns	ns	ns
CIV vs FBN	ns	ns	ns
CIV vs FCL	ns	ns	ns
CIV vs **GG**	ns	****	ns
**CIV** vs LN	ns	ns	**
CN vs FBN	ns	ns	ns
CN vs FCL	ns	ns	ns
CN vs **GG**	*	**	ns
**CN** vs LN	ns	***	****
FBN vs FCL	ns	ns	ns
FBN vs **GG**	ns	****	ns
**FBN** vs LN	ns	ns	****
FCL vs **GG**	ns	*	ns
**FCL** vs LN	****	***	****
**GG** vs LN	****	****	****

ZO-1 Continuous

Comparisons	0cAMP	1cAMP	3cAMP
CIV vs CN	ns	ns	ns
CIV vs FBN	ns	ns	ns
CIV vs FCL	ns	ns	ns
CIV vs **GG**	ns	****	ns
**CIV** vs LN	ns	ns	***
CN vs FBN	ns	ns	ns
CN vs FCL	ns	ns	ns
CN vs **GG**	ns	***	ns
**CN** vs LN	ns	**	****
FBN vs FCL	ns	ns	ns
FBN vs **GG**	ns	****	ns
**FBN** vs LN	ns	ns	****
FCL vs **GG**	ns	**	ns
**FCL** vs LN	****	**	****
**GG** vs LN	****	****	****

VE-Cad Coverage

Comparisons	0cAMP	1cAMP	3cAMP
CIV vs CN	ns	ns	ns
CIV vs **FBN**	ns	ns	***
CIV vs FCL	ns	ns	ns
CIV vs **GG**	ns	****	**
CIV vs LN	ns	ns	ns
CN vs **FBN**	ns	ns	**
CN vs **FCL**	*	ns	ns
CN vs **GG**	ns	****	*
**CN** vs LN	ns	****	ns
FBN vs **FCL**	**	ns	ns
FBN vs **GG**	ns	****	ns
**FBN** vs LN	ns	**	****
FCL vs **GG**	ns	****	ns
**FCL** vs LN	*	****	**
**GG** vs LN	ns	****	****

VE-Cad Continuous

Comparisons	0cAMP	1cAMP	3cAMP
CIV vs CN	ns	ns	ns
CIV vs **FBN**	ns	ns	**
CIV vs FCL	ns	ns	ns
CIV vs **GG**	ns	****	*
CIV vs LN	ns	ns	ns
CN vs **FBN**	ns	ns	**
CN vs FCL	ns	ns	ns
CN vs **GG**	ns	****	*
**CN** vs LN	ns	**	ns
**FBN** vs FCL	**	ns	ns
**FBN** vs GG	ns	****	ns
**FBN** vs LN	ns	*	****
FCL vs **GG**	ns	****	ns
**FCL** vs LN	*	***	**
**GG** vs LN	ns	****	****

ZO-1 Punctate

Comparisons	0cAMP	1cAMP	3cAMP
CIV vs CN	ns	ns	ns
CIV vs FBN	ns	ns	ns
CIV vs FCL	ns	ns	ns
CIV vs GG	ns	ns	ns
**CIV** vs LN	**	ns	ns
CN vs FBN	ns	ns	ns
CN vs **FCL**	**	ns	ns
CN vs **GG**	**	ns	ns
CN vs LN	ns	ns	ns
FBN vs FCL	ns	ns	ns
FBN vs GG	ns	ns	ns
**FBN** vs LN	**	ns	ns
FCL vs GG	ns	ns	ns
**FCL** vs LN	****	ns	ns
**GG** vs LN	****	ns	ns

ZO-1 Perpendicular

Comparisons	0cAMP	1cAMP	3cAMP
CIV vs CN	ns	ns	ns
CIV vs FBN	ns	ns	ns
CIV vs FCL	ns	ns	ns
CIV vs GG	ns	ns	ns
CIV vs LN	ns	ns	ns
**CN** vs FBN	ns	ns	*
CN vs FCL	ns	ns	ns
**CN** vs GG	ns	ns	*
CN vs LN	ns	ns	ns
FBN vs **FCL**	ns	ns	**
FBN vs GG	ns	ns	ns
FBN vs **LN**	ns	ns	****
**FCL** vs GG	ns	ns	**
FCL vs LN	ns	ns	ns
GG vs **LN**	ns	*	***

VE-Cad Punctate

Comparisons	0cAMP	1cAMP	3cAMP
CIV vs CN	ns	ns	ns
CIV vs FBN	ns	ns	ns
CIV vs FCL	ns	ns	ns
**CIV** vs GG	ns	****	ns
CIV vs LN	ns	ns	ns
**CN** vs FBN	ns	ns	*
CN vs FCL	ns	ns	ns
**CN** vs GG	ns	***	ns
CN vs LN	ns	ns	ns
FBN vs FCL	ns	ns	ns
FBN vs GG	ns	ns	ns
FBN vs LN	* (Fbn)	ns	*(LN)
**FCL** vs GG	ns	****	ns
FCL vs LN	ns	ns	ns
GG vs **LN**	ns	****	ns

VE-Cad Perpendicular

Comparisons	0cAMP	1cAMP	3cAMP
CIV vs CN	ns	ns	ns
CIV vs FBN	ns	ns	ns
CIV vs FCL	ns	ns	ns
CIV vs GG	ns	ns	ns
CIV vs LN	ns	ns	ns
CN vs FBN	ns	ns	ns
**CN** vs FCL	ns	****	ns
CN vs GG	ns	ns	ns
**CN** vs LN	ns	**	ns
FBN vs FCL	ns	ns	ns
FBN vs GG	ns	ns	ns
FBN vs LN	ns	ns	ns
FCL vs GG	ns	ns	ns
FCL vs LN	ns	ns	ns
GG vs LN	ns	ns	ns

Table S4. Statistical Analysis for Junction Phenotyping of 4-day culture. Comparison between each substrate protein, with 0d, 1d, or 3d cAMP treatment is presented, as calculated using the Kruskal-Wallis test with a Dunn's multiple comparison test. A red box marked with "ns" signifies $p > 0.05$. A green box signifies a significant difference, where * $p < 0.05$, ** $p < 0.01$, *** $p < 0.001$, and **** $p < 0.0001$, and bold text (or parentheses, when needed) indicates which protein generated higher coverage. Data corresponds to Supplemental Figure S7.

Figure S8 – Part 1.

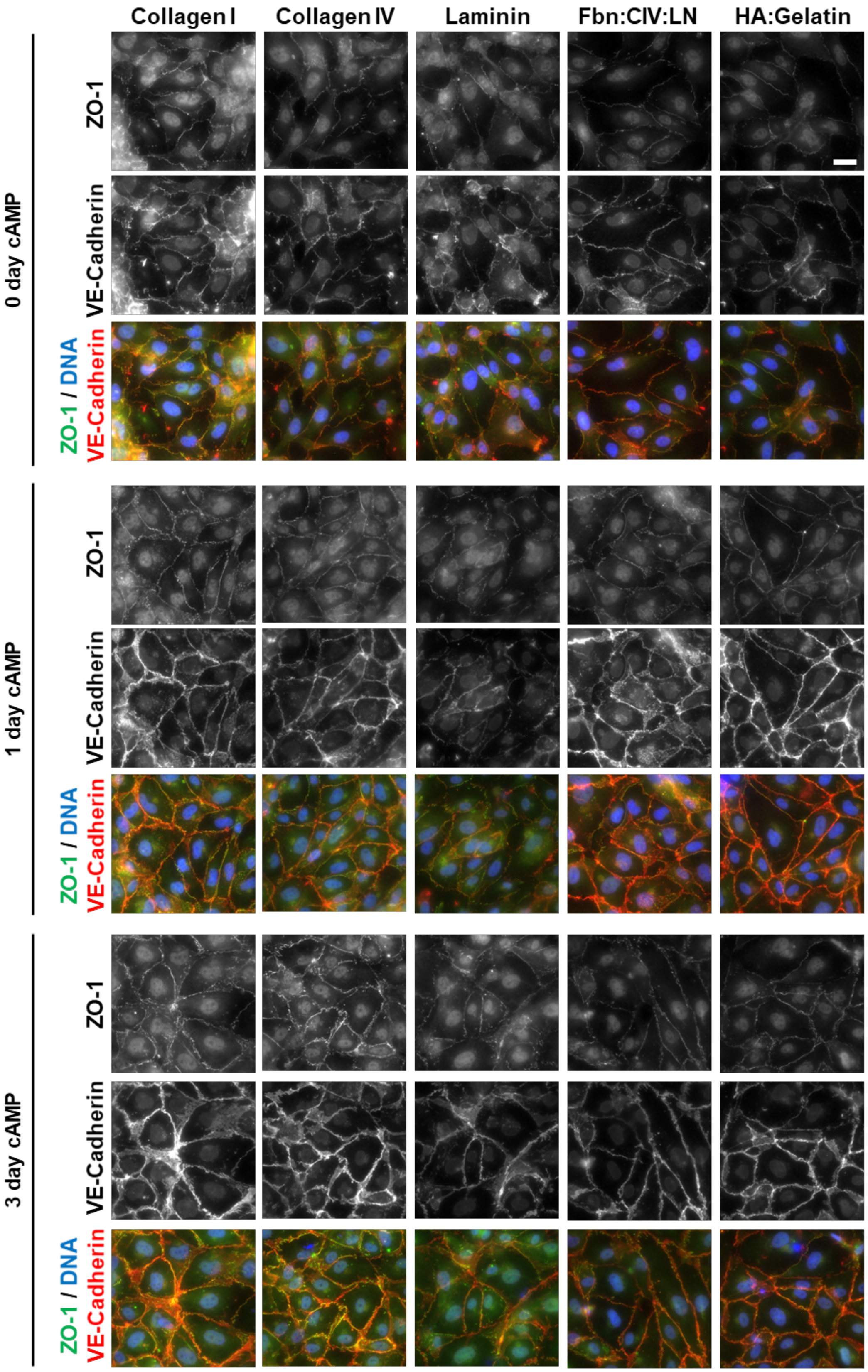

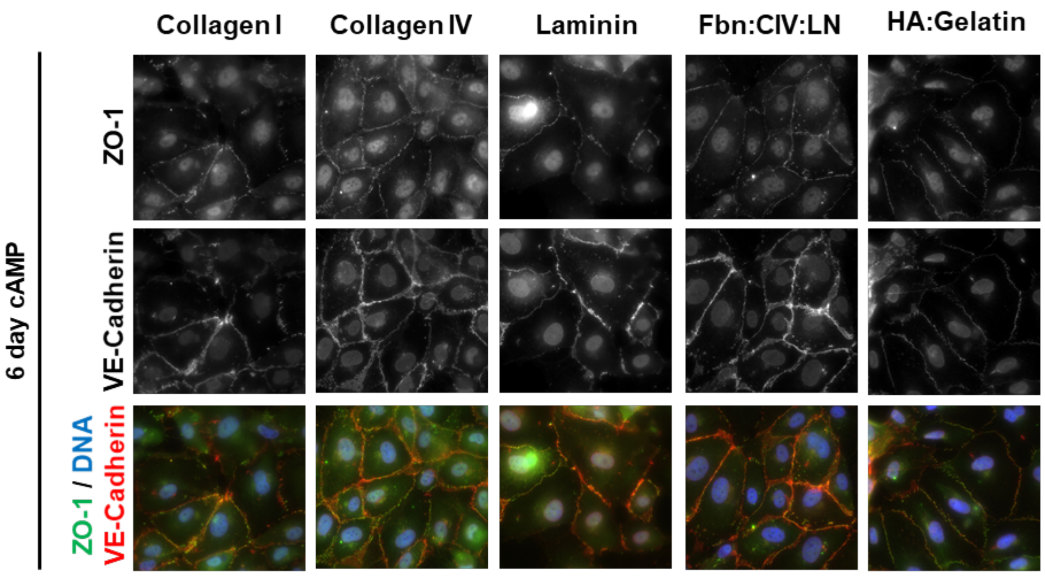

Figure S8. Immunofluorescence Images of HBMECs in 7-day Culture. HBMECs on 5 substrate coatings, cultured for 7 days, with 0d, 1d, 3d, or 6d cAMP treatment. Stained for ZO-1 (green), VE-cadherin (red), and DNA (blue). (scale bar = 20 μm)

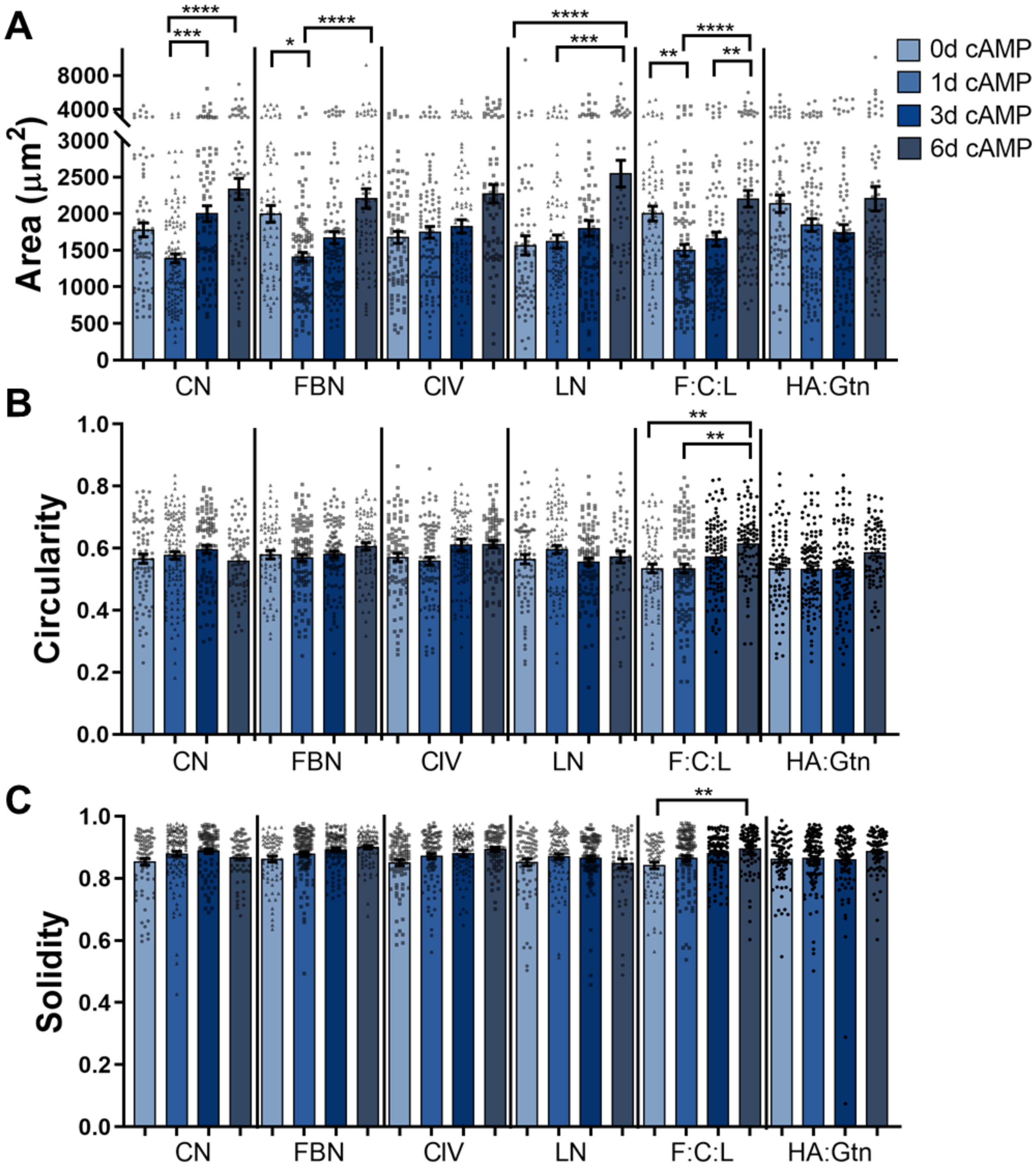

Figure S9. Cell Morphology Analysis for 7-day Culture. Cell area (A), circularity (B), and solidity (C) of HBMECs cultured on the 6 substrate coatings for 7 days, with 0d, 1d, 3d, or 6d cAMP treatment. $56 \leq N \leq 126$, where N is the number of cells. The Kruskal-Wallis test with a Dunn's multiple comparison test was used to calculate significant differences, where* $p < 0.05$, ** $p < 0.01$, *** $p < 0.001$, and **** $p < 0.0001$. See Supplemental Table S5 for comparative statistical analysis between protein coatings.

Cell Area

Comparisons	0cAMP	1cAMP	3cAMP	6cAMP
CIV vs CN	ns	ns	ns	ns
CIV vs FBN	ns	ns	ns	ns
CIV vs FCL	ns	ns	ns	ns
CIV vs GG	ns	ns	ns	ns
CIV vs LN	ns	ns	ns	ns
CN vs FBN	ns	ns	ns	ns
CN vs FCL	ns	ns	ns	ns
CN vs **GG**	ns	*	ns	ns
CN vs LN	ns	ns	ns	ns
FBN vs FCL	ns	ns	ns	ns
FBN vs GG	ns	ns	ns	ns
FBN vs LN	ns	ns	ns	ns
FCL vs GG	ns	ns	ns	ns
**FCL** vs LN	*	ns	ns	ns
**GG** vs LN	*	ns	ns	ns

Cell Circularity

Comparisons	0cAMP	1cAMP	3cAMP	6cAMP
CIV vs CN	ns	ns	ns	ns
CIV vs FBN	ns	ns	ns	ns
CIV vs FCL	ns	ns	ns	ns
CIV vs GG	ns	ns	ns	ns
CIV vs LN	ns	ns	ns	ns
CN vs FBN	ns	ns	ns	ns
CN vs FCL	ns	ns	ns	ns
CN vs GG	ns	ns	ns	ns
CN vs LN	ns	ns	ns	ns
FBN vs FCL	ns	ns	ns	ns
FBN vs GG	ns	ns	ns	ns
FBN vs LN	ns	ns	ns	ns
FCL vs GG	ns	ns	ns	ns
FCL vs LN	ns	ns	ns	ns
GG vs **LN**	ns	*	ns	ns

Cell Solidity

Comparisons	0cAMP	1cAMP	3cAMP	6cAMP
CIV vs CN	ns	ns	ns	ns
CIV vs FBN	ns	ns	ns	ns
CIV vs FCL	ns	ns	ns	ns
CIV vs GG	ns	ns	ns	ns
CIV vs LN	ns	ns	ns	ns
CN vs FBN	ns	ns	ns	ns
CN vs FCL	ns	ns	ns	ns
CN vs GG	ns	ns	ns	ns
CN vs LN	ns	ns	ns	ns
FBN vs FCL	ns	ns	ns	ns
FBN vs GG	ns	ns	ns	ns
FBN vs LN	ns	ns	ns	ns
FCL vs GG	ns	ns	ns	ns
FCL vs LN	ns	ns	ns	ns
GG vs LN	ns	ns	ns	ns

Table S5. Statistical Analysis for Cell Morphology of 7-day Culture. The comparison between each substrate protein with 0d, 1d, 3d and 6d cAMP is presented, as calculated using the Kruskal-Wallis test with a Dunn's multiple comparison test. A red box marked with "ns" signifies $p > 0.05$. A green box signifies a significant difference, where * $p < 0.05$ and ** $p < 0.01$ and bold text indicates which protein generated the higher value. Data corresponds to Supplemental Figure S9.

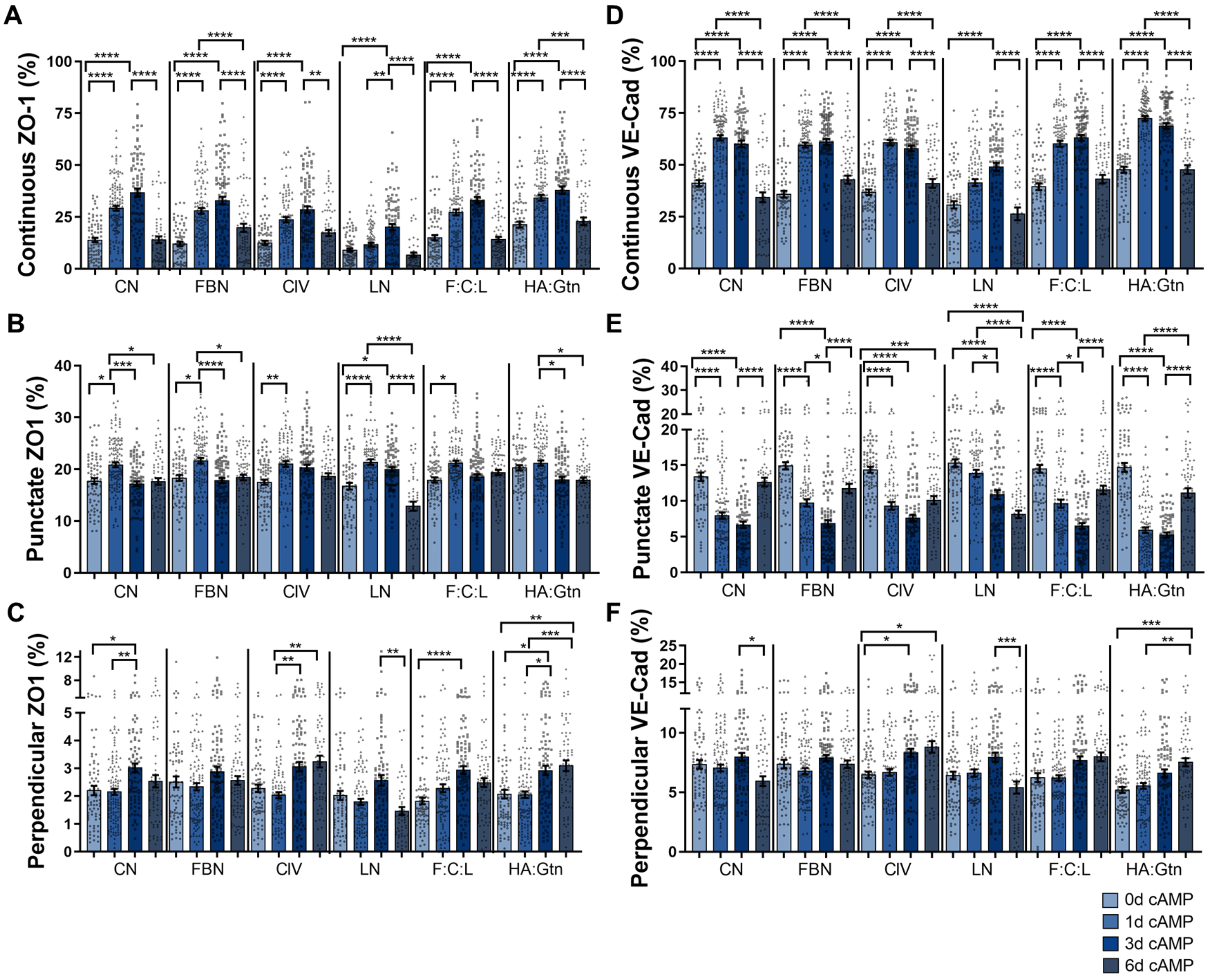

Figure S10. Junction Phenotype Analysis for 7-day Culture. Edge presentation of continuous (A), punctate (B), and perpendicular (C) junctions for for ZO-1 (A-C) and VE-cadherin (D-F). $56 \leq N \leq 126$, where N is the number of cells. The Kruskal-Wallis test with a Dunn's multiple comparison test was used to calculate significant differences, where * $p < 0.05$, ** $p < 0.01$, *** $p < 0.001$, and **** $p < 0.0001$. See Supplemental Table S6 for comparative statistical analysis between protein coatings.

Table S6. Statistical Analysis for Junction Phenotyping of 7-day culture. The comparison between each substrate protein, with 0d, 1d, 3d, or 6d cAMP treatment is presented, as calculated using the Kruskal-Wallis test with a Dunn's multiple comparison test. A red box marked with "ns" signifies $p > 0.05$. A green box signifies a significant difference, where * $p < 0.05$, ** $p < 0.01$, *** $p < 0.001$, and **** $p < 0.0001$, and bold text indicates which protein generated higher coverage. Data corresponds to Supplemental Figure S10.

ZO-1 Coverage

Comparisons	0cAMP	1cAMP	3cAMP	6cAMP
CIV vs CN	ns	ns	ns	ns
CIV vs FBN	ns	ns	ns	ns
CIV vs FCL	ns	ns	ns	ns
CIV vs **GG**	***	***	ns	ns
**CIV** vs LN	ns	****	ns	****
CN vs FBN	ns	ns	ns	ns
CN vs FCL	ns	ns	ns	ns
CN vs **GG**	*	ns	ns	ns
**CN** vs LN	ns	****	ns	ns
FBN vs FCL	ns	ns	ns	ns
FBN vs **GG**	**	ns	ns	ns
**FBN** vs LN	ns	****	**	****
FCL vs GG	ns	ns	ns	ns
**FCL** vs LN	ns	****	****	**
**GG** vs LN	****	****	****	****

ZO-1 Continuous

Comparisons	0cAMP	1cAMP	3cAMP	6cAMP
CIV vs CN	ns	ns	ns	ns
CIV vs FBN	ns	ns	ns	ns
CIV vs FCL	ns	ns	ns	ns
CIV vs **GG**	*	**	ns	ns
**CIV** vs LN	ns	****	ns	**
CN vs FBN	ns	ns	ns	ns
CN vs FCL	ns	ns	ns	ns
CN vs GG	ns	ns	ns	ns
**CN** vs LN	ns	****	****	ns
FBN vs FCL	ns	ns	ns	ns
FBN vs **GG**	*	ns	ns	ns
**FBN** vs LN	ns	****	***	****
FCL vs GG	ns	ns	ns	ns
**FCL** vs LN	ns	****	****	ns
**GG** vs LN	****	****	****	****

ZO-1 Punctate

Comparisons	0cAMP	1cAMP	3cAMP	6cAMP
**CIV** vs CN	ns	ns	*	ns
CIV vs FBN	ns	ns	ns	ns
CIV vs FCL	ns	ns	ns	ns
CIV vs GG	ns	ns	ns	ns
**CIV** vs LN	ns	ns	ns	**
CN vs FBN	ns	ns	ns	ns
CN vs FCL	ns	ns	ns	ns
CN vs GG	ns	ns	ns	ns
CN vs LN	ns	ns	ns	ns
FBN vs FCL	ns	ns	ns	ns
FBN vs GG	ns	ns	ns	ns
FBN vs LN	ns	ns	ns	ns
FCL vs GG	ns	ns	ns	ns
**FCL** vs LN	ns	ns	ns	****
**GG** vs LN	*	ns	ns	*

ZO-1 Perpendicular

Comparisons	0cAMP	1cAMP	3cAMP	6cAMP
CIV vs CN	ns	ns	ns	ns
CIV vs FBN	ns	ns	ns	ns
CIV vs FCL	ns	ns	ns	ns
CIV vs GG	ns	ns	ns	ns
**CIV** vs LN	ns	ns	ns	****
CN vs FBN	ns	ns	ns	ns
CN vs FCL	ns	ns	ns	ns
CN vs GG	ns	ns	ns	ns
CN vs LN	ns	ns	ns	ns
FBN vs FCL	ns	ns	ns	ns
FBN vs GG	ns	ns	ns	ns
**FBN** vs LN	ns	ns	ns	***
FCL vs GG	ns	ns	ns	ns
**FCL** vs LN	ns	ns	ns	**
**GG** vs LN	ns	ns	ns	****

VE-Cad Coverage

Comparisons	0cAMP	1cAMP	3cAMP	6cAMP
CIV vs CN	ns	ns	ns	ns
CIV vs FBN	ns	ns	ns	ns
CIV vs FCL	ns	ns	ns	ns
CIV vs **GG**	*	**	*	ns
**CIV** vs LN	ns	****	ns	ns
CN vs FBN	ns	ns	ns	ns
CN vs FCL	ns	ns	ns	ns
CN vs GG	ns	ns	ns	ns
**CN** vs LN	ns	****	ns	ns
FBN vs FCL	ns	ns	ns	ns
FBN vs **GG**	ns	***	ns	ns
**FBN** vs LN	ns	****	*	ns
FCL vs **GG**	ns	***	ns	ns
**FCL** vs LN	ns	****	***	*
**GG** vs LN	***	****	ns	***

VE-Cad Continuous

Comparisons	0cAMP	1cAMP	3cAMP	6cAMP
CIV vs CN	ns	ns	ns	ns
CIV vs FBN	ns	ns	ns	ns
CIV vs FCL	ns	ns	ns	ns
CIV vs **GG**	ns	**	**	ns
**CIV** vs LN	ns	****	ns	ns
CN vs FBN	ns	ns	ns	ns
CN vs FCL	ns	ns	ns	ns
CN vs **GG**	ns	*	ns	ns
**CN** vs LN	ns	****	*	ns
FBN vs FCL	ns	ns	ns	ns
FBN vs **GG**	ns	****	ns	ns
**FBN** vs LN	ns	****	**	ns
FCL vs **GG**	ns	***	ns	ns
**FCL** vs LN	ns	****	****	ns
**GG** vs LN	ns	****	****	**

VE-Cad Punctate

Comparisons	0cAMP	1cAMP	3cAMP	6cAMP
CIV vs CN	ns	ns	ns	ns
CIV vs FBN	ns	ns	ns	ns
CIV vs FCL	ns	ns	ns	ns
**CIV** vs GG	ns	**	ns	ns
CIV vs **LN**	ns	****	*	ns
CN vs FBN	ns	ns	ns	ns
CN vs FCL	ns	ns	ns	ns
CN vs GG	ns	ns	ns	ns
CN vs **LN**	ns	****	***	**
FBN vs FCL	ns	ns	ns	ns
**FBN** vs GG	ns	****	ns	ns
FBN vs **LN**	ns	****	****	ns
**FCL** vs GG	ns	***	ns	ns
FCL vs **LN**	ns	****	****	ns
GG vs **LN**	ns	****	****	ns

VE-Cad Perpendicular

Comparisons	0cAMP	1cAMP	3cAMP	6cAMP
**CIV** vs CN	ns	ns	ns	***
CIV vs FBN	ns	ns	ns	ns
CIV vs FCL	ns	ns	ns	ns
CIV vs GG	ns	ns	ns	ns
**CIV** vs LN	ns	ns	ns	****
CN vs FBN	ns	ns	ns	ns
CN vs **FCL**	ns	ns	ns	*
**CN** vs GG	**	ns	ns	ns
CN vs LN	ns	ns	ns	ns
FBN vs FCL	ns	ns	ns	ns
**FBN** vs GG	**	ns	ns	ns
**FBN** vs LN	ns	ns	ns	*
FCL vs GG	ns	ns	ns	ns
**FCL** vs LN	ns	ns	ns	***
**GG** vs LN	ns	ns	ns	**

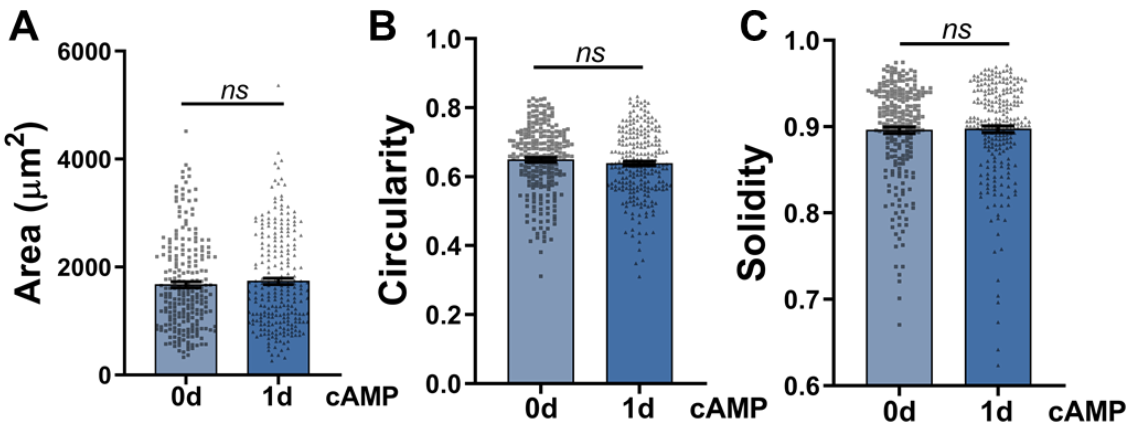

Figure S11. Cell Morphology for Transwell Permeability Assay. Cell area (A), circularity (B), and solidity (C) of HBMECs cultured on Transwell inserts coated with FBN, cultured for 2 days, with 0d or 1d cAMP treatment. $53 \leq N \leq 72$, where N is the number of cells. The Mann-Whitney test was used to calculate significant differences, where ns = $p > 0.05$, * $p < 0.05$, ** $p < 0.01$, *** $p < 0.001$ and **** $p < 0.0001$.

Additional Method S1. Trans-Endothelial Electrical Resistance (TEER) Assay. For the TEER assay, cells were cultured on Transwell inserts (24-well) with a 0.4 µm pore size (Falcon, 353047) according to the 4-day treatment schedule (Figure 3.A). On Day 0, the inserts were coated with 100 µg/ml FBN and incubated for 1 hour at 37 °C. Excess solution was then removed, and the inserts were rinsed with warm PBS (+/+). The top and bottom chambers of the system were then respectively filled with 100 µl and 800 µl of warm HBMEC medium and placed in the incubator during cell splitting. HBMECs were then plated at $5x10^4$ cells/cm^2 and the volume of the top chamber was then brought to 200 µl. The controls for this experiment were a blank insert and an insert with just the FBN coating for each condition. Starting on Day 1, resistance measurements were performed using an EVOM^2 meter and performed every day for the duration of the experiment. Electrodes were rinsed in warm PBS (+/+) then HBMEC medium prior to measurement of each sample. The days on which medium was changed, the TEER measurement was performed prior to the media change. After collecting the measurement on Day 4, the inserts were rinsed with warm PBS then fixed as described in the methods section. Prior to imaging, the membranes were removed from the insert, inverted, and sandwiched between two glass coverslips with PBS.

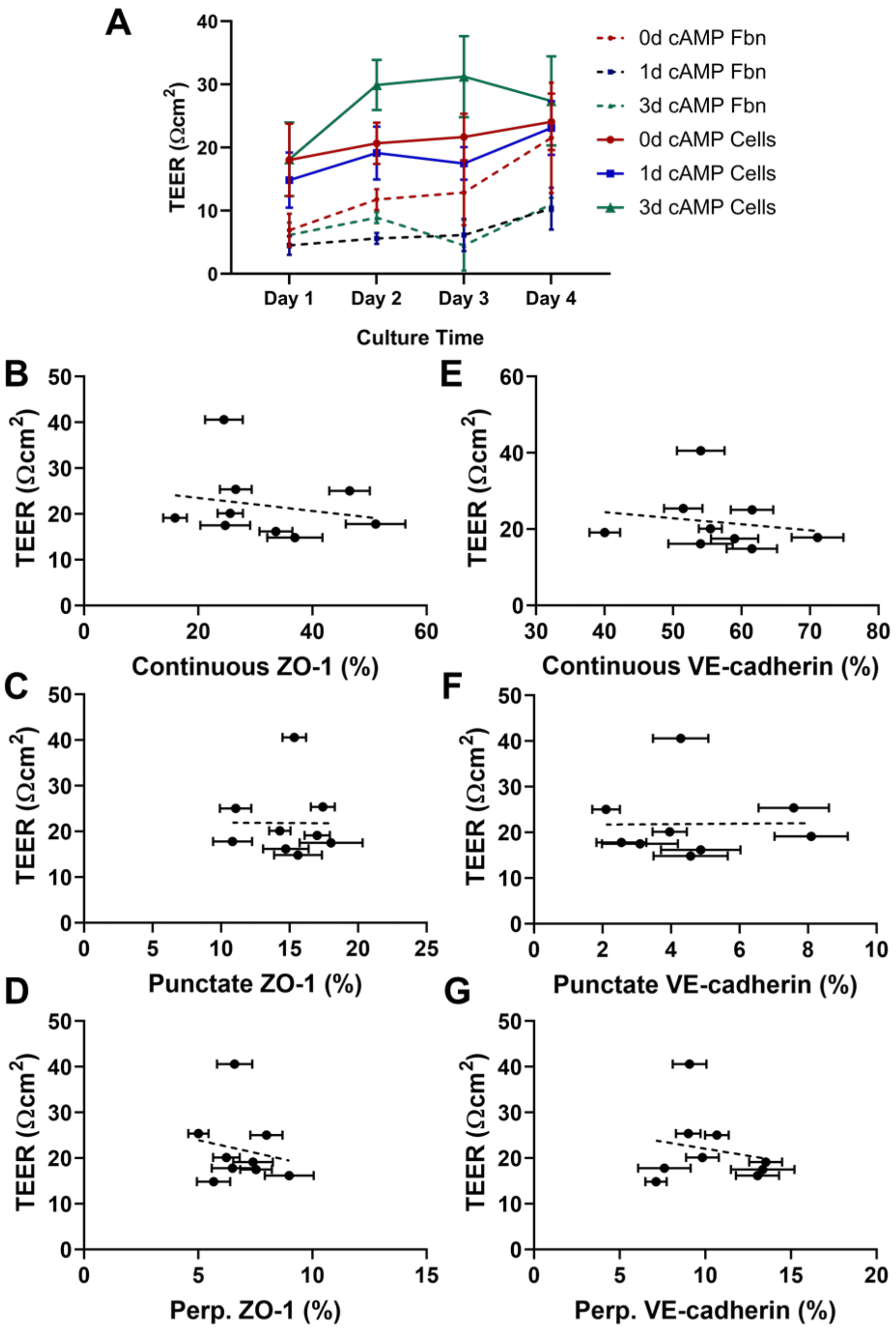

Figure S12. TEER Assay and Junction Phenotype Correlation. (A) Resistance measurements of HBMECs cultured on FBN-coated Transwell inserts for 4 days with 0d, 1d, or 3d cAMP treatment. Control measurements without cells are also presented. N = 3, where N= number of trials. A two-way ANOVA indicated significant differences for each condition versus their FBN-only control , presented in the Figure legend, where * $p < 0.05$ and **** $p < 0.0001$. Correlation of each junction type for ZO-1 (B-D) and VE-cadherin (E-G) coverage with TEER, where a linear regression rendered the slope of all relationships non-significantly non-zero. N = 9, where N is the number of inserts pooled between 3 trials of the 0d, 1d, and 3d cAMP conditions.

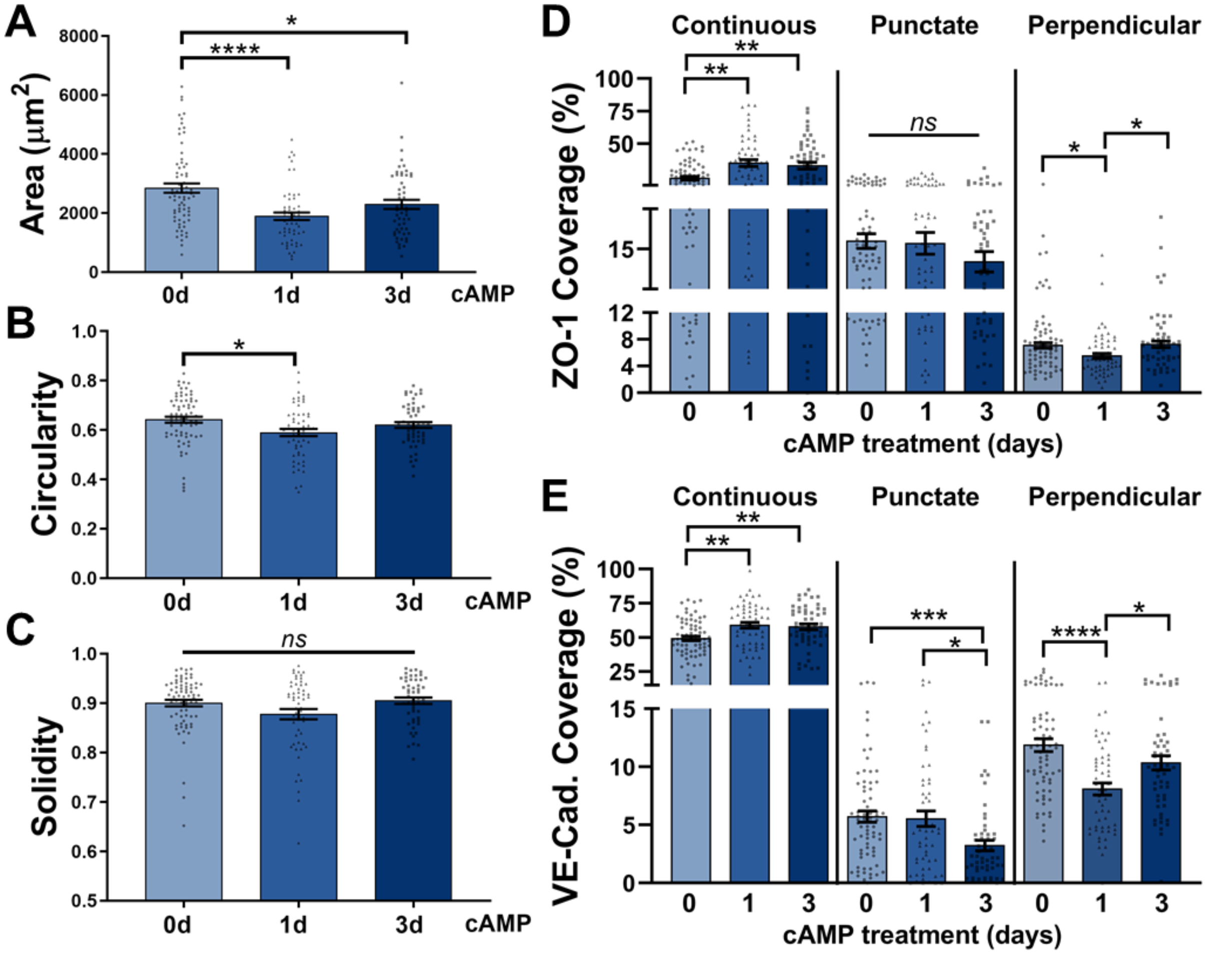

Figure S13. Cell Morphology and Junction Phenotyping from TEER Assay. Cell area (A), circularity (B), and solidity (C) of HBMECs cultured on Transwell inserts coated with FBN, cultured for 4 days, with 0d, 1d, and 3d cAMP treatment. Edge presentation of continuous, punctate, and perpendicular junctions for ZO-1 (D) and VE-cadherin (E). $53 \leq N \leq 72$, where N is the number of cells. The Kruskal-Wallis test with a Dunn's multiple comparison test was used to calculate significant differences for each parameter, where * $p < 0.05$, ** $p < 0.01$, *** $p < 0.001$ and **** $p < 0.0001$.

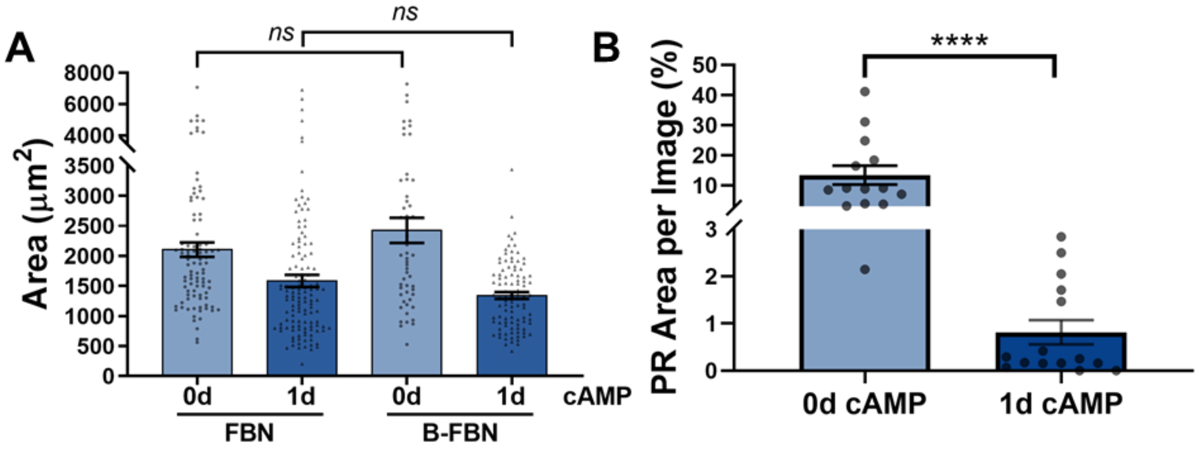

Figure S14. Local Permeability Assay. (A) Comparison of cell area for HBMECs on FBN versus B-FBN with and without cAMP. 53 ≤ N ≤ 125, where N is the number of cells pooled between 3 trials. (B) Effect of cAMP on PR area. The percentage of each image area containing areas of PR. N = 15, where N is the number of images (5 images per trial, 3 trials). The Mann-Whitney test was used to calculate significance, where **** p < 0.0001.

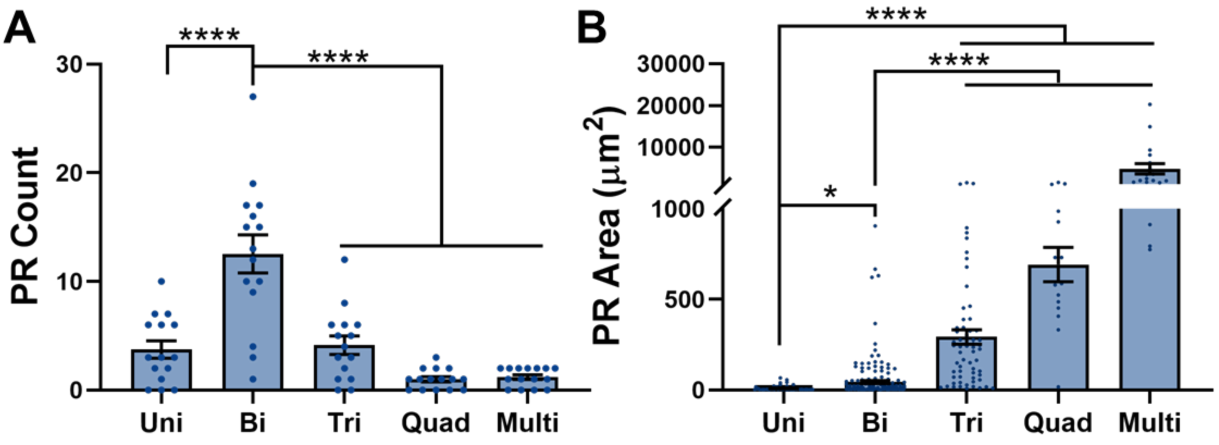

Figure S15. PR Analysis based on ZO-1 Images. The average number of each PR type per image is presented in (A) while the average size of each PR type is presented in (B). N = 15 for (A) where N is the number of images. 15 ≤ N ≤ 189 for (B) where N is the number of PRs. The Kruskal-Wallis test with a Dunn's multiple comparison test was used to calculate significant differences, where **** p < 0.0001.

	% Junction		# Junction	
	ZO-1	VE-Cad.	ZO-1	VE-Cad.
Cont.	ns	ns	****	****
No Junct.	ns	ns	****	****
Disc.	*	ns	****	****
Punct.	*	ns	****	****
Perp.	ns	ns	****	****

Table S7. Statistical Analysis for Slope Deviation from Zero for Junction Presentation versus PR Area. The analysis of junction type for ZO-1 and VE-cadherin, as calculated using Linear Regression. A red box marked with "ns" signifies $p > 0.05$. A green box signifies a significant difference, where * $p < 0.05$ and **** $p < 0.0001$. Data corresponds to Figure 9.

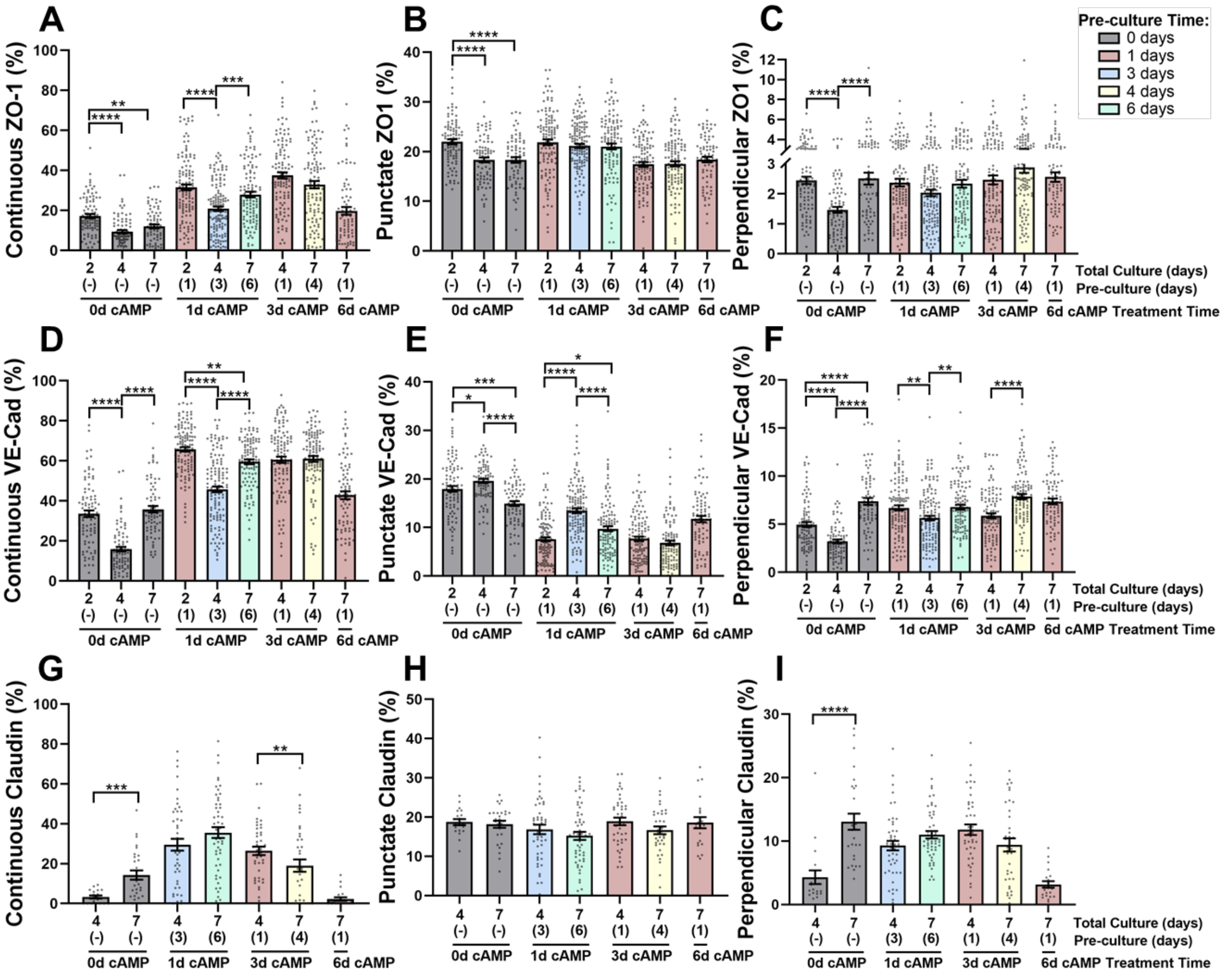

Figure S16. Effect of total culture and pre-culture time on junction phenotype on FBN. Edge presentation of continuous (left panels), punctate (middle panels), and perpendicular (right panels) junctions for ZO-1 (A-C), VE-cadherin (D-F), and Claudin (G-I). Labels indicate the total time in culture (top row), the time in culture prior to cAMP treatment (i.e. pre-culture time, middle row and bar color), and the time of cAMP treatment bottom row. $74 \leq N \leq 145$ for (A-D) and $19 \leq N \leq 52$ for (E-F), where N is the number of cells. Statistical analysis was used to compare results within the same cAMP treatment group to directly compare the effect of pre-culture time. The Kruskal-Wallis test with a Dunn's multiple comparison test was used to calculate significant differences for groups of at least 3 comparisons and a Mann-Whitney test was used to compare groups of 2. Significance is represented by ** $p < 0.01$, *** $p < 0.001$, and **** $p < 0.0001$. Data re-plotted from Figures 2-5.
